# Self-Assembly Protein Superstructures as a Powerful Chemodynamic Therapy Nanoagent for Glioblastoma Treatment

**DOI:** 10.1007/s40820-020-00490-6

**Published:** 2020-07-15

**Authors:** Tao Zheng, Wentao Wang, Jon Ashley, Ming Zhang, Xiaotong Feng, Jian Shen, Yi Sun

**Affiliations:** 1 Department of Health Technology, Technical University of Denmark, 2800 Kongens Lyngby, Denmark; 2 Jiangsu Collaborative Innovation Center for Biomedical Functional Materials, School of Chemistry and Materials Science, Nanjing Normal University, Nanjing, 210023 People’s Republic of China; 3 Key Laboratory of High Performance Polymer Material and Technology of Ministry of Education, School of Chemistry and Chemical Engineering, Nanjing University, Nanjing, 210023 People’s Republic of China

**Keywords:** Self-assembly protein superstructures, Glioblastoma therapy, Chemodynamic therapy, Self-delivery entities, Blood–brain barrier

## Abstract

Glioblastoma (GBM) remains a formidable challenge in oncology. Chemodynamic therapy (CDT) that triggers tumor cell death by reactive oxygen species (ROS) could open up a new door for GBM treatment. Herein, we report a novel CDT nanoagent. Hemoglobin (Hb) and glucose oxidase (GOx) were employed as powerful CDT catalysts. Instead of encapsulating the proteins in drug delivery nanocarriers, we formulate multimeric superstructures as self-delivery entities by crosslinking techniques. Red blood cell (RBC) membranes are camouflaged on the protein superstructures to promote the delivery across blood–brain barrier. The as-prepared RBC@Hb@GOx nanoparticles (NPs) offer superior biocompatibility, simplified structure, and high accumulation at the tumor site. We successfully demonstrated that the NPs could efficiently produce toxic ROS to kill U87MG cancer cells in vitro and inhibit the growth of GBM tumor in vivo, suggesting that the new CDT nanoagent holds great promise for treating GBM.

**Electronic supplementary material:**

The online version of this article (10.1007/s40820-020-00490-6) contains supplementary material, which is available to authorized users.

## Highlights

- A new type of protein nanostructure as a chemodynamic therapy nanoagent was fabricated via proper assembling and crosslinking techniques.
- The as-fabricated nanostructures could cross the blood–brain barrier, possessing excellent long circulating times and accumulation properties at the tumor site.
- The RBC@Hb@GOx NPs can be regarded as ‘pure particles of drugs,’ which can produce toxic reactive oxygen species to inhibit the growth of orthotopic brain tumor.

**Electronic supplementary material:**

The online version of this article (10.1007/s40820-020-00490-6) contains supplementary material, which is available to authorized users.

## Graphical abstract

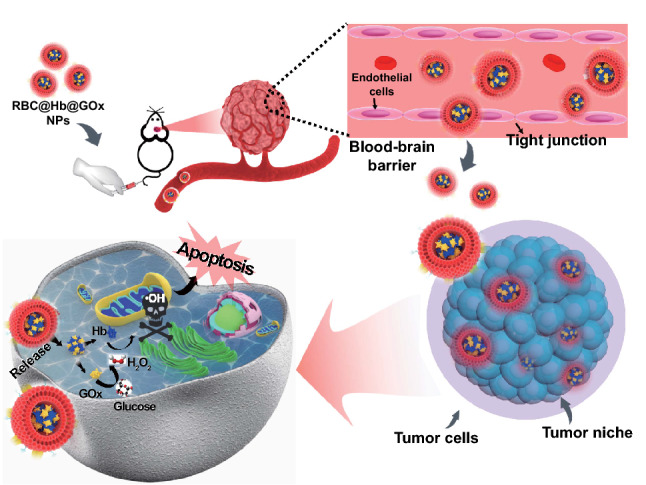

## Introduction

Glioblastoma multiforme (GBM) is the most malignant cancer in our central nervous system (CNS) [1]. It has an incidence of three per 100,000 adults per year and accounts for 52% of all primary brain tumors. The mainstay of treatment is surgery [2], followed by radiation and chemotherapy [3, 4]. Due to the aggressive nature and the localization in brain, standard therapies are ineffective in managing the devastating disease. Most GBM tumors are infiltrating and grow into the normal brain tissues, making it difficult to eliminate the cancerous tissues by surgery [5]. The efficacy of radiotherapy and chemotherapy is largely limited by the phenotypic and genotypic heterogeneity, hypoxic tumor microenvironment (TME), and intrinsic cell resistance mechanisms [6]. Translation of immunotherapy to GBM is also a distinct challenge, as the CNS is traditionally considered immune privileged [7]. With a 5-year survival rate of < 10% and median survival of only 15 months, GBM remains a formidable challenge in the field of oncology [8]. Developing new treatments for GBM will be of high importance for both the patients and the healthcare system.

Recently, chemodynamic therapy (CDT) has emerged as a new reactive oxygen species (ROS)-mediated therapeutic strategy. It employs Fenton-type reactions to convert intracellular hydrogen peroxide (H_2_O_2_) into highly cytotoxic hydroxyl radial (·OH), triggering the death of cancer cells through the apoptosis or necrosis processes [9–13]. With the ability to modulate ROS in TME, CDT might open up new opportunities for GBM treatment. Nevertheless, despite the fact that various sophisticated CDT nanoagents have been engineered, CDT is still in its infancy and has seldom been used in brain cancers. A number of critical issues including toxicity of the nanomaterials, complexity of the structures, and difficulty in penetrating BBB must be addressed before applying CDT in clinical practice.

To realize CDT, it is important to select materials with excellent Fenton catalytic activity [14, 15]. Up to now, mainly metal-based inorganic materials and metal–organic framework (MOF) have been exploited as catalysts [16, 17]. Unfortunately, safety remains the predominant concern for the CDT nanoagents. It is known that metallic nanoparticles are prone to elicit toxic responses in the brain. Shao et al. concluded that the autophagy, inflammatory response, and disturbed signaling pathways might be the main mechanisms underlying the neurotoxicity of metallic nanoparticles [18]. Moreover, at physiological conditions, most organic/inorganic Fenton catalysts are non-biodegradable. They may persist in the body for long periods, causing systematic toxicity due to prolonged exposure to different biological microenvironments. Thus, it is imperative to create highly biocompatible and biodegradable Fenton catalysts to minimize nerotoxicity and systemic toxicity.

Besides the toxicity, another challenge of the CDT nanoagent is their complex structures. Typical CDT nanoagents consist of three major components: Fenton catalysts, nanocarriers, and modifiers/targeting ligands coated on outer surface. Several nanocarriers made of a zeolitic imidazolate framework [19, 20], block copolymers [21–23], or dendritic mesoporous silica [24, 25] have been devised to encapsulate Fenton catalysts. These fancy materials and structures demonstrated the advances in nanotechnology. However, the complicated formulations, tedious synthesis processes, and instability of the colloids have become the major hurdles in the clinical translation of CDT. Therefore, useful CDT nanoagents must possess characteristics of ease of synthesis, simplified structures, and enhanced stability.

In addition to the drawbacks of the CDT nanoagents, developing CDT for GBM treatment is further hampered by the presence of the highly restrictive blood–brain tumor barrier and blood–brain tumor barrier (BBTB) [26, 27]. The BBB/BBTB can limit the penetration of therapeutics from the systemic circulation into the tumor compartment [28]. One popular approach to facilitate the drug delivery across BBB/BTB is receptor-mediated transcytosis, which hijacks the receptors/transporters expressed on the barriers [29, 30]. Different ligands have been reported to actively target the transferrin receptors, insulin-like growth factors [31], or low-density lipoprotein receptors [32]. However, these receptors are ubiquitously expressed in several cell types and tissues, leading to unwanted peripheral organ uptake. Moreover, the transport capacity of endocytosis is rather low due to the limited number of membrane transport proteins and the complex intracellular trafficking procedures. The best ligand published so far only delivered less than 1% of the injected dose to brain, with even less ending up at the tumor site [33]. As such, increasing the accumulation of Fenton agents at GBM would greatly improve the outcome of CDT.

**Fig. 1:**
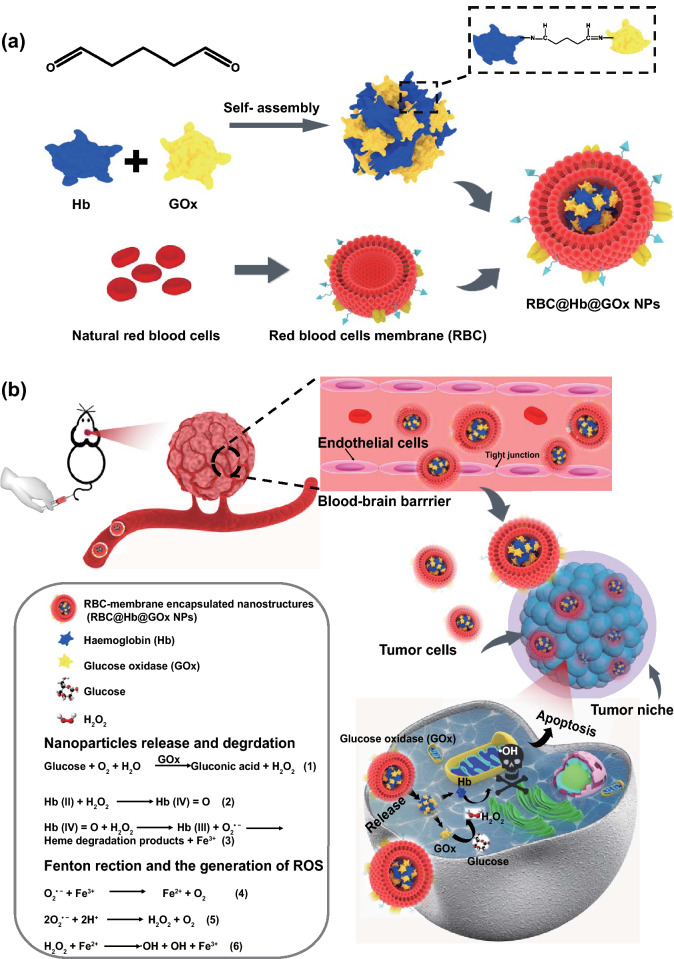
Design of hemoglobin, gluocose oxidase–based protein superstructures as a powerful chemodynamic therapy nanoagent for glioblastoma treatment. **a** Schematic illustration of synthesizing RBC@Hb@GOx NPs. **b** Scheme of TME-activated ROS production by RBC@Hb@GOx NPs for GBM treatment

In this study, to tackle the major obstacles in CDT, we constructed a new type of CDT nanoagent (Fig. 1a). The potential toxicity of inorganic nanomaterials and MOFs have urged us to turn our attention to the plethora of natural proteins. In fact, a large number of proteins in nature, the so-called metalloproteins, contain metal ion cofactors, which can act as Fenton catalytic ions, such as Fe^2+^, Cu^2+^, Mn^2+^, and Co^2+^. Examples include Hb [34] nitrite reductase [35] and ceruloplasmin [36]. In addition, some proteins are able to generate exogenous H_2_O_2_, such as GOx [37] and NADPH oxidase [38]. In this study, we showed that the combination of Hb and GOx could be an attractive alternative to the synthetic Fenton catalysts. The predominant advantages offered by the natural proteins are the high biocompatibility and biodegradability. Employing them for CDT significantly improves the safety of the therapy.

To co-deliver the two proteins-Hb and GOx, instead of loading them into the nanocarriers, we proposed a new concept of ‘self-delivery.’ With proper assembling and crosslinking techniques, the two protein monomers were formulated into multimeric protein superstructures (denoted as Hb@GOx NPs), thus eliminating the need of the nanocarrier component in conventional drug delivery systems. The protein superstructures present a unique way to deliver multiple proteins. They can be regarded as ‘pure particles of drugs,’ which greatly simplifies the structure and the synthesis steps and offers extremely high drug loading yields. In addition, the stability of the protein nanoparticles is much more enhanced compared to free proteins loosely encapsulated in the carriers.

To facilitate the delivery of Hb@GOx NPs to the GBM microenvironment, we employed a passive targeting strategy to cross BBB/BBTB. It has been shown that for disorders such as GBM, the integrity of the BBB is partially compromised. Hence, passive targeting is possible if the size of NPs is sufficiently small and the circulation time is long. We camouflaged the protein superstructures with red blood cell (RBC) membranes (denoted as RBC@Hb@GOx NPs). The CD 47 protein and glycosyl groups on the membrane surface endow the nanoparticles with prolonged systematic retention time, less reticuloendothelial system uptake, and reduced immuno-recognition [39, 40]. Compared to the active targeting, passive targeting would lead to enhanced delivery efficiency.

The working mechanism of the novel CDT nanoagent is shown in Fig. 1b. The RBC@Hb@GOx NPs are administrated intravenously and circulate with the blood. The RBC membrane helps with passive diffusion across the endothelial cells of BBB/BBTB. Once reaching the tumor site, the nanoagents are uptaken by the tumor cells. The abundant in situ H_2_O_2_ causes cell membrane destruction, which increases the access of Hb@GOx protein superstructures. The two electron oxidation of Fe(II)–Hb by H_2_O_2_ produces the Fe(IV)-ferrylHb that can react with an additional molecule of H_2_O_2_, resulting in the degradation of heme groups and the release of free ions. The ions catalyze Fenton reactions, converting H_2_O_2_ to toxic ·OH and O_2_^·−^ (Fig. 1b, Eqs. 1–6). In the meantime, GOx consumes oxygen and glucose to generate H_2_O_2_ and gluconic acid. The increased acidity and H_2_O_2_ in the microenvironment further boost the Fenton reactions. Eventually, the protein superstructures are degraded, and the tumor cells are killed by the excessive oxidative stress. In this paper, we successfully proved that the novel RBC@Hb@GOx NPs could inhibit the growth of GBM in mice models. To our best knowledge, this is the first time that the CDT nanoagent was constructed with 100% natural biomaterials and the in vivo therapeutic efficacy of CDT was demonstrated in GBM.

## Experimental Section

### Preparation of Hb@GOx NPs

Hb powder (25 mg) and GOx (15 mg) powder were added to MQ water (6 mL) and stirred overnight at 500 rpm using a magnetic stirrer for complete hydration. Afterward, 10 µL of 2% glutaraldehyde solution was mixed to induce intra-particle crosslinking. The solution was stirred continuously at 500 rpm at room temperature for 24 h.

### Preparation of ICG-Loaded Hb@GOx NPs

Hb powder (25 mg), GOx (15 mg), and 6 mg ICG powder (6 mg) were added to MQ water (6 mL) and then were stirred overnight at 500 rpm using a magnetic stirrer for complete hydration. The following process was the same as mentioned above.

### Preparation of RBCs Membrane

Briefly, the blood was taken from the cattle and diluted by PBS (pH 7.4). After centrifuging for 5 min (3000 rpm, 4 °C), the collected RBCs were washed 3 times (pH 7.4), re-dispersed in the water and placed in the ice bath for 50 min. Finally, the solution was centrifuged (12,000 rpm, 4 °C) and then washed with PBS (pH 7.4) until the supernatant became colorless. The obtained RBCs membrane was re-dispersed in PBS (pH 7.4), sonicated, and filtered through a 0.45 μm and 0.22 μm filter before use.

### Preparation of RBC Membrane Modifying Hb@GOx or ICG-Loaded Nanoparticles

100 µL of RBC membrane was added to 400 µL of MQ water. The solution was sonicated for 10 s before being mixed with 500 µL of Hb@GOx NPs or ICG-loaded Hb@GOx NPs. The mixture solution was sonicated for 60 s to complete the membrane coating. The excess RBC membrane was removed by centrifugation, and RBCs@Hb@GOx NPs or ICG-RBCs@Hb@GOx NPs were re-dispersed in MQ water for further use.

### Evaluation the Generation of H_2_O_2_ In Vitro

The catalytic performance of RBCs@Hb@GOx NPs in the presence of glucose (10 mM) was conducted using fluorimetric hydrogen peroxide assay kit. The produced H_2_O_2_ generates a red fluorescent product (λ_ex_ = 540/λ_em_ = 590 nm). At each time point (0, 10, 20, 30, and 45 min), the production of H_2_O_2_ was calculated according to the fluorescence standard curve obtained from a fluorescent microplate reader. In addition, the UV–Vis absorbance of the mixed solution at 425 nm was measured to further investigate the degradation of RBCs@Hb@GOx NPs at room temperature.

### Detection of Hydroxyl Radicals In Vitro

The 1,3-diphenylisobenzonfuran (DPBF, Sigma-Aldrich), as a hydroxyl radical indicator, was applied to investigate the generation of hydroxyl radicals via measuring the absorption intensity at wavelength of 410 nm in UV–Vis absorption spectrum. Firstly, the test solution (RBCs@Hb@GOx NPs mixed with glucose) was prepared, and samples were collected at reaction time points of 0, 10, 15, 20, and 25 min. After that, DPBF (100 μL) in methanol (20 mM) was added into the sample solutions (100 μL). The absorption intensity of DPBF was recorded. At same time, the group of DPBF solution treated with Hb plus H_2_O_2_ solution was set as the control group.

### Electron Spin Resonance (ESR) Measurements for the Detection of Hydroxyl Radicals (·OH) and Superoxide Radical (O_2_^·−^) In Vitro

For detecting ·OH detection, BMPO (25 mM, 50 μg mL^−1^) in PBS, Hb@GOx NPs, and RBCs@Hb@GOx NPs mixed with glucose (10 mM) or without glucose (as control group) were prepared. ESR spectra were recorded after incubation of 6 min. For detecting O_2_^·−^, BMPO (25 mM, 50 μg mL^−1^) in PBS, Hb@GOx NPs and RBCs@Hb@GOx NPs mixed with glucose (10 mM) or without glucose (as control group) were prepared. ESR spectra were recorded after incubation of 6 min.

### Cell Culture

NIH3T3 and U87MG cell lines were obtained from the American Type Culture Collection (ATCC). All cell lines were stored in a 100% humidity atmosphere of 37 °C and 5% CO_2_.

### In Vitro Cytotoxicity Assay

MTT assay was applied to evaluate the cytotoxicity of RBCs@Hb@GOx NPs. Firstly, NIH3T3 or U87MG cells were randomly seeded into 96-well plates and incubated with RBCs@Hb@GOx NPs at different concentrations (0, 50, 100, 200, 300, 400, 500, 600, 700, 800, and 1000 μg mL^−1^) at 100% humidity atmosphere at 37 °C and 5% CO_2_ for 24 h. Then, 20 μL of MTT solution was added into each well, placed in CO_2_ incubator for further incubation for 4 h. DMSO (150 μL) was added, and the absorbance at 490 nm was obtained to calculate cell survival rate by using MTT assay kit.

### Hemolysis Assay

Firstly, the obtained cattle blood red cells were washed with PBS by centrifuging. Next, the properly diluted blood samples were added into the RBCs@Hb@GOx NPs solution with different sample concentrations (500 − 2500 μg mL^−1^). After being stationary in PBS (negative controls) and deionized water (positive controls) for 1 h, the supernatant was obtained after centrifuging and was further accessed to calculate the hemolysis ratio.

### In Vitro Chemodynamic Therapy for U87MG Cells

U87MG cells (1 × 10^4^ cells per well) were seeded into a 96-well and incubated with PBS, Hb, GOx, Hb@GOx NPs, RBCs@Hb@GOx NPs, Hb and H_2_O_2_, GOx and H_2_O_2_, Hb@GOx NPs and H_2_O_2_, RBC@Hb@GOx NPs and H_2_O_2_, Hb and glucose, GOx and glucose, Hb@GOx NPs and glucose, RBC@Hb@GOx NPs and glucose at 100% humidity atmosphere of 37 °C and 5% CO_2_ for 24 h, respectively. Next, 20 μL of MTT solution was added into each well and placed in CO_2_ incubator for further incubating for 4 h. DMSO (150 μL) was added, and the absorbance at 490 nm was obtained to calculate cell survival rate using the MTT assay kit. Moreover, in order to investigate the chemodynamic efficacy of RBC@Hb@GOx NPs on U87MG cells in vitro, Calcein-AM (100 μL) and PI solution (100 μL) were incubated with U87MG cells for 30 min. Living cells were stained with calcein-AM (green fluorescence) and dead cells with PI (red fluorescence).

### Evaluation of the Production of Intracellular ROS

The U87MG cells were randomly seeded into 6-well plates and then incubated with different groups under DMEM at a 100% humidity atmosphere at 37 °C and 5% CO_2_ for 24 h. After that, the cells were washed with PBS (centrifuged at 1000 rpm for 5 min), adjusting the cell concentration to 1 × 10^6^ mL^−1^. DCFH-DA was diluted with serum-free culture solution at 1:1000 to achieve a final concentration of 10 μM. Then, the collected cells were suspended in diluted DCFH-DA solution and further incubated at 37 °C for 20 min. Besides, the incubation solution was shaken to make the probe fully contact with the cell. Finally, the cells were washed with serum-free cell culture medium 3 times to fully remove DCFH-DA that did not enter into cells. The fluorescence intensity of cells was determined by confocal fluorescence microscope.

Quantitative analyses for ROS production were analyzed by flow cytometric (Becton–Dickinson FACS Calibur) experiments. The U87MG cells after being treated with different groups were incubated with 10 μM of DCFH-DA for 20 min. Then, the incubation solutions were analyzed by flow cytometry with excitation at 488 nm and emission at 530 nm.

### Evaluation of Mitochondrial Damage In Vitro

The U87MG cells were randomly seeded into 6-well plates and then incubated with different groups under 90% DMEM plus 10% FBS at a 100% humidity atmosphere of 37 °C and 5% CO_2_ for 24 h. The logarithmic growth cells were seeded into six-well plate. When the cells grew adhering to the wall after 24 incubation, the corresponding drug-containing medium was added according to the group setting, and a negative control group was set up. Then, the cells were washed with PBS (centrifuged at 2000 rpm for 5 min), adjusting the cell concentration to 1 × 10^6^ mL^−1^. Next, the 100 μL of 10 × buffer solution and 900 μL of sterilized deionized water were diluted into 1 × Buffer, mixed and preheated to 37 °C. JC-1 working solution was formed via absorbing 500 μL of incubation buffer (1 × Buffer) into 1 μL of JC-1 solution. After that, 500 μL of JC-1 working solution was suspended with cells and incubated them at 37 °C and 5% CO_2_ incubator for 15–20 min. Finally, cells treated with different groups were collected by centrifugation at room temperature (1000 rpm, 5 min) and washed twice in 1 × Buffer, and further analyzed by confocal fluorescence microscope and flow cytometry.

### HMGB1 Release Assay

U87MG cells were seeded into in 6-well plates. After 24-h treatment with different groups, dead cells were removed by centrifugation and the supernatants were concentrated to 100 μL. Released HMGB1 in supernatants were tested via western blot.

### Preparing Mouse Model

U87MG human glioma cells were purchased from American Type Culture Collection (ATCC) and maintained in DMEM media (Gibco) with 10% FBS (Hyclone). Six-week-old NCR nude female mice were used to generate intracranial orthotopic U87MG gliomas. Briefly, mice were anesthetized using 2% isoflurane and their heads were immobilized in a stereotactic headframe using atraumatic ear bars. A burr hole was made using a steel drill bit (Plastics One, Roanoke, VA, USA) 1.4 mm right of the sagittal and 1 mm anterior to the lambdoid suture. Tumors were allowed to grow for 14 days prior to treatment. Intracranial tumor growth was monitored in vivo using bioluminescence IVIS^®^ imaging (Xenogen, Almeda, CA) equipped with LivingImage™ software (Xenogen).

### Evaluation of Transportability across the BBB In Vivo

ICG-loaded Hb@GOx NPs or RBC@Hb@GOx NPs were employed to investigate the transportability across the BBB based on the ICG fluorescence at tumor site. The orthotopic U87MG gliomas (without self-fluorescence) bearing mice were intravenously injected with ICG-Hb@GOx NPs and ICG-RBC@Hb@GOx NPs (250 μL). The fluorescence signal at brain was detected in vivo using bioluminescence IVIS^®^ imaging equipped with LivingImage™ software (Xenogen) at 0, 6, 12, 36, 48, and 72 h post-injection. In addition, the main organs from the treated mice, including heart, liver, spleen, lung, kidney, and brain, were collected after 72 h injection for imaging and biodistribution analysis.

### Evaluation of Anti-tumor Efficiency In Vivo

The orthotopic U87MG gliomas (with self-fluorescence) bearing mice were randomly divided into four groups: (1) PBS (as control group, 200 mL); (2) GOx (250 μL, 40 mg kg^−1^); (3) Hb@GOx NPs (250 μL, 40 mg kg^−1^); and (4) RBC@Hb@GOx NPs (250 μL, 40 mg kg^−1^). Treatment began from day 21 after tumor implantation, and all treatments were intravenously administered. They were administered once every 10 days. After treatment with above groups, tumor response to treatment was tracked using IVIS imaging. Signal intensity was quantified within a region of interest using Living Image™ software.

### In Vivo Biocompatibility Evaluation

The slice from major organs and tumor was obtained from each group after 40 days. H&E staining was conducted for histological examination and observed via Olympus BX43 microscope (Japan).

### TUNEL, H&E and Congo Red Staining

TUNEL Apoptosis Assay was used for determining tumor cells apoptosis. The orthotopic U87MG gliomas bearing mice brain tissue were stained with TUNEL Apoptosis Assay Kit after treatment. For histology, the frozen tissue sections of 4 ~ 5μm were immersed in 1% acetone fixation solution at room temperature for 5 min. Afterwards the tissue sections were dried, washed with PBS (3 times) for 3 min, and further stained with H&E and Congo Red. Finally, the collected sections were analyzed by an Olympus BX43 microscope (Japan). All staining experiments were conducted following the standard protocol.

### Statistical Analysis

The statistical analyses were conducted by Student’s *t* test. Differences were considered statistically significant at *p* < 0.05.

### Ethical Approval

All experiments involving animals were performed in compliance with relevant ethical regulations in adherence with the Nanjing University and Nanjing Normal University for the Care and Use of Laboratory Animals, Nanjing, China.

## Results and Discussion

### Characterization of Hb@GOx NPs and RBC@Hb@GOx NPs

**Fig. 2:**
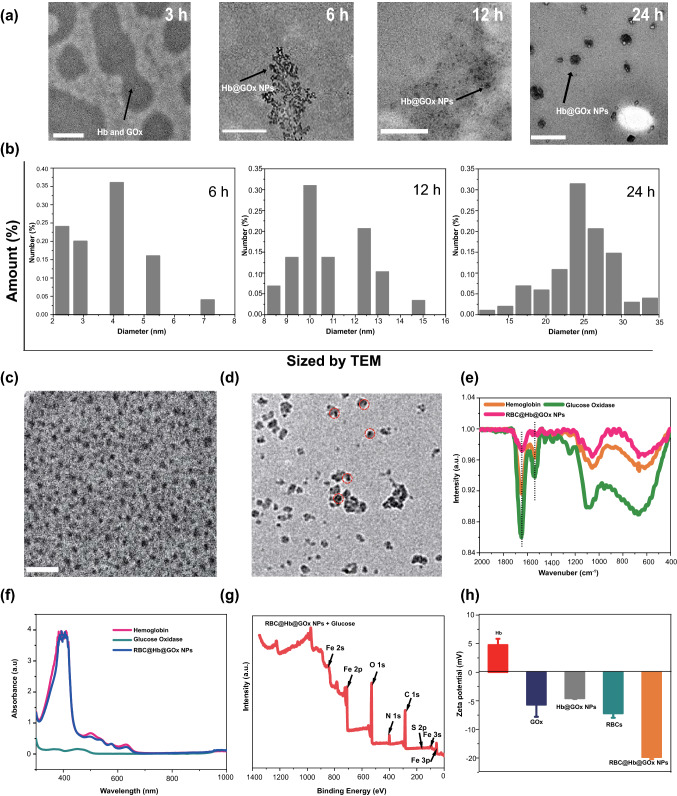
Characterization of as-fabricated Hb@GOx NPs and RBC@Hb@GOx NPs. **a** TEM images of as-fabricated Hb@GOx NPs at each reaction time. **b** Size of Hb@GOx NPs at each reaction time measured by TEM. Scale bar: 100 nm. TEM images of **c** Hb@GOx NPs (scale bar: 100 nm) and **d** RBC@ Hb@GOx NPs (scale bar: 100 nm). **e** FT-IR spectra of Hb, GOx and RBC@Hb@GOx NPs. **f** UV–Vis absorption spectra of free Hb, GOx and RBC@Hb@GOx NPs. **g** XPS of RBC@Hb@GOx NPs. **h** Zeta potentials of the Hb, GOx, RBC membranes, Hb@GOx NPs and RBC@Hb@GOx NPs. Data are shown as mean ± SD (*n* = 3)

The key technical challenge here is to develop an appropriate method to construct the protein superstructures, so that their biological activities can be retained. There have been crosslinked protein nanoparticles derived from gelatin [41], albumin [42], and gliadin [43]. The existing fabrication methods such as coacervation and desolvation usually unfold the proteins to decrease the intramolecular hydrophobic interactions. They are not applicable as the functionality of the proteins is destroyed during the process. In this work, we developed a facile ‘dissolving-crosslinking’ approach to prepare the protein particulates under mild conditions without the use of harsh chemicals or organic solvents. The process for synthesizing Hb@GOx NPs and RBC@Hb@GOx NPs is shown in Figs. 1a and S1. Briefly, Hb and GOx powder were added into MQ water, and then, the mixed solution was stirred overnight for complete hydration. Afterward, glutaraldehyde (GA) was added as crosslinker to form intermolecular interactions and induce the assembly of Hb and GOx. The low-degree crosslinking caused little loss to the bioactivities of the proteins. To investigate the whole assembly process and determine the size of as-fabricated nanoparticles, the dynamic light scattering (DLS) and transmission electron microscopy (TEM) were used to monitor the reaction at four time points (3, 6, 12, and 24 h). With the increase in the reaction time, the NPs became bigger and more homogenous, as shown in Fig. 2a. We chose 24 h as the crosslinking time due to the uniform morphology observed via TEM. The representative TEM shows that the as-fabricated Hb@GOx NPs possess an average size of 34.03 nm (Fig. 2b, c) with good polydispersity index (PDI) of 0.435 (Fig. S2a). After camouflaged by the RBC membrane, the average size increased to 51.55 nm (Figs. 2d and S2b).

The Fourier transform infrared (FT-IR) spectrum is shown in Fig. 2e. The shapes of the infrared absorption bands of amide I and amide II in Hb molecule provided detailed information on the secondary structure of the polypeptide chain. The absorption band between 1700 and 1600 cm^−1^ belonging to amide I was attributed to C=O stretching vibration of peptide linkages in the protein’s backbone. The absorption band of 1620–1500 cm^−1^ for amide II was due to a combination of N–H bending and C–N stretching. Normally, the absorption bands of amide I and amide II are eliminated when the structure of Hb is denatured. Figure 2e displays the FT-IR spectra of Hb, GOx, and RBC@Hb@GOx NPs. The absorption bands for amide I and amide II in the RBC@Hb@GOx NPs were located at 1643.91 and 1536.78 cm^−1^, respectively, which were nearly the same as those obtained for the native protein (1650.01 and 1536.79 cm^−1^) [44] The absorption bands of the RBC@Hb@GOx NPs in amide I drifted slightly from reference values, indicating an interaction between Hb and GOx. In addition, the absorption peaks at around 450 nm of RBC@Hb@GOx NPs in UV–Vis absorption spectrum was the same as Hb (Fig. 2f), suggesting that the structure of Hb was well maintained.

The X-ray photoelectron spectroscopy (XPS) was applied to analyze the existing elements in the as-prepared NPs. No obvious Fe peaks were detected from the samples without adding glucose solution (Fig. S3), indicating the tight combination between the Fe^2+^ and heme. After incubation with glucose solution, Fenton reactions occurred. The peaks belonging to Fe 2*p* and Fe 3*p* were detected (Fig. 2g), indicating that the Fe^2+^ was released from heme group (Fig. S4). Besides, the presence of peaks of Fe 2*p*, Fe 3*p*, Fe 2*s*, and Fe 3*s* in the high-resolution XPS further verified the existence of Fe^2+^ and Fe^3+^ in the Fenton reaction (Fig. S5). The zeta potential was measured after each synthesis procedure, with a value of 4.79 ± 1.05 mV for Hb, −5.70 ± 2.09 mV for GOx, −4.59 ± 0.1 mV for Hb@GOx, −7.22 ± 0.75 mV for RBCs, and −14.867 ± 0.33 mV for RBC@Hb@GOx NPs (Figs. 2h and S6). Finally, western blotting (WB) assay was applied to further confirm the existence of the CD47 membrane proteins. It was found CD47 protein was still retained on RBCs@Hb@GOx NPs (Fig. S7), validating the successful coating of erythrocyte membrane.

### Evaluation of Catalytic Performance of RBC@Hb@GOx NPs

**Fig. 3:**
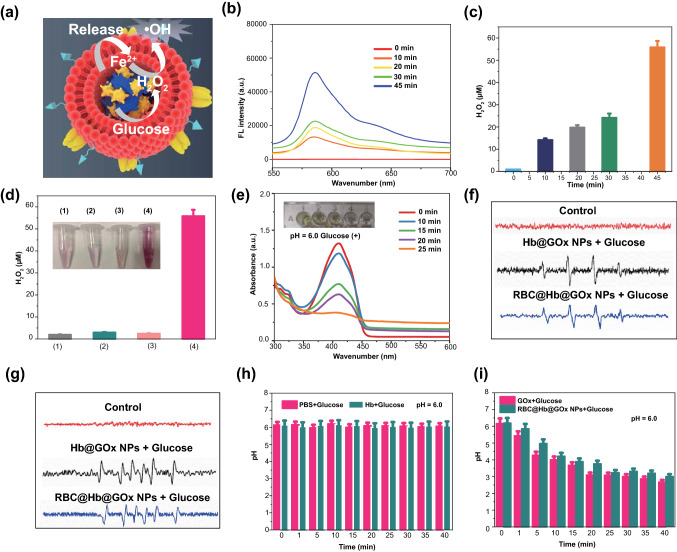
Verification of H_2_O_2_ and ROS (·OH, O_2_^·−^) generation in vitro. **a** Synergism schematic of Fenton reaction of RBC@Hb@GOx NPs triggered by tumor microenvironment. **b** Fluorescence spectra of Amplex red probe incubated with RBC@Hb@GOx NPs in the presence 10 mM of glucose. **c** Production of H_2_O_2_ during the catalytic reactions. **d** Amplex red probe incubated with different groups (PBS, Hb, RBC@Hb@GOx NPs without glucose, and RBC@Hb@GOx NPs with glucose). **e** UV–Vis absorption spectrum of DPBF. **f**, **g** ESR spectra of BMPO after incubation of Hb@GOx NPs and RBC@Hb@GOx NPs with glucose (10 mM). **h** pH values of PBS, Hb in 10 mM of glucose. **i** pH values of Hb and GOx, and RBC@Hb@GOx NPs in 10 mM of glucose. Data are shown as mean ± SD (*n* = 3)

The Fenton catalytic performance of the RBC@Hb@GOx NPs was evaluated [45, 46]. The RBC@Hb@GOx NPs were incubated with glucose solution (10 mM) to start cascade Fenton-type reactions (Figs. 3a and S8). Amplex red, a fluorescence probe that can respond to H_2_O_2_ with fluorescence intensity changes, was applied to measure the generated H_2_O_2_ during the catalytic process. As presented in Fig. 3b, c, the fluorescence intensity of Amplex red increased significantly with the reaction time, indicating the efficient production of H_2_O_2_. Almost no H_2_O_2_ were observed in control groups (Fig. 3d), suggesting that the H_2_O_2_ was produced by GOx-medicated glucose oxidation. To detect the production of free radicals, 1,3-diphenylisobenzofuran (DPBF) was employed to quantitatively analyze ·OH [47]. The generated ·OH can oxidize DPBF and result in the reduction in the absorption intensity at wavelength of 410 nm in UV–Vis absorption spectrum. In Fig. 3e, the absorption at 410 nm of DPBF decreases obviously after 25 min reaction. The results provided a clear evidence that the bioactivities of the GOx and Hb were not affected after the crosslinking reactions.

Since electron spin resonance (ESR) spectrum has been considered as the most effective assay to detect the generation of ROS [48]. We selected 5-tertbutoxycarbonyl-5-methy-1-pyrroline N-oxide (BMPO), the traditional ROS trapping agents, to confirm the generation of ·OH and O_2_^·−^. As shown in Fig. 3f, g, both Hb@GOx NPs and RBC@Hb@GOx NPs triggered the Fenton reactions in glucose solution (10 mM) and resulted in two typical spectra. The four-peak spectrum with an intensity ratio of 1:2:2:1 belonged to the BMPO/OH adduct, while the spectrum with an intensity ratio of 1:1:1:1 showed adduct of BMPO/O_2_^·−^. Thus, as-fabricated NPs had excellent performance to produce ·OH and O_2_^·−^.

We then studied the change in pH values during the catalytic reaction. As shown in Fig. 3h, i, for RBC@Hb@GOx NPs incubated with glucose, the pH value continually decreased from 6.20 to 3.19 within 45 min due to the generation of gluconic acid. The experimental results further validated that GOx’s remained original activity remained after crosslinking with Hb. It is worth noting that there are synergic effects by combining Hb and GOx for Fenton reactions. It is well known that TME is hypoxic, which limits the performance of GOx since the oxygen is critical in catalyzing glucose into H_2_O_2_. This problem was perfectly overcome by Hb, which is an oxygen-carrying protein. The oxygen-carrying capability of Hb was investigated in Fig. S9, showing that the dissolved oxygen in RBC@Hb@GOx solution increased gradually with time. In short, our results suggested that the RBC@Hb@GOx NPs could be utilized as a powerful CDT nanoagent.

### Evaluation of In Vitro Cytotoxicity of RBC@Hb@GOx NPs

**Fig. 4:**
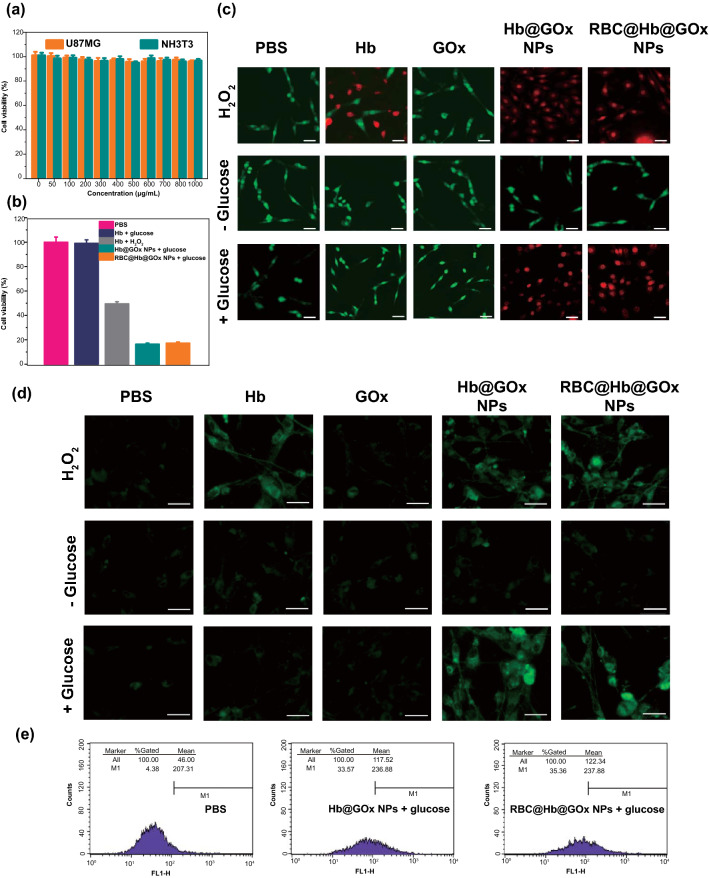
In vitro cytotoxicity and intracellular catalytic mechanism. **a** Cytotoxicity of RBC@Hb@GOx NPs to U87MG and NIH3T3 cells without glucose. **b** Cell viability assay of (1) PBS, (2) Hb and glucose (10 mM), (3) GOx and glucose, (4) Hb and H_2_O_2_, (5) GOx and H_2_O_2_, (6) Hb@GOx NPs and glucose, and (7) RBC@Hb@GOx NPs and glucose-treated U87MG cells. **c** Live/dead assay of U87MG cells after incubation with different samples for 24 h and subsequent staining with AM/PI probe. Scale bar: 100 μm. **d** Detection of intracellular ROS after incubating U87MG with different samples. Scale bar: 50 μm. **e** The level of intracellular ROS was measured by flow cytometry using the peroxide-sensitive fluorescent probe DCFH-DA. Data are shown as mean ± SD (*n* = 3)

To explore the in vitro cytotoxicity of RBC@Hb@GOx NPs, methyl thiazolyl tetrazolium (MTT) assays were conducted [49]. U87MG and NHI3T3 cells were incubated with RBC@Hb@GOx NPs with concentrations ranging from 0 to 1000 ug mL^−1^ (Fig. 4a). RBC@Hb@GOx NPs showed negligible cytotoxicity in the cell lines, even at the concentration as high as 1000 ug mL^−1^. In addition, the acquired hemolysis ratio was much less than 1% when the concentration of RBC@Hb@GOx NPs reached 2500 μg mL^−1^ (Fig. S10). Moreover, the degradation of RBC@Hb@GOx NPs was also investigated via UV–Vis absorption spectrum (Fig. S11). The NPs completely degraded after 30 min in glucose solution. These results indicated that the nanoparticles possess excellent biocompatibility and biodegradability. When the glucose was added to a mixture of cells and RBC@Hb@GOx NPs, the NPs were activated. High toxicity to the cells was observed as a result of ROS-caused oxidative damage (Fig. 4b). In comparison, adding glucose to Hb solution did not have a marked effect on the viability due to the lack of GOx to convert glucose to H_2_O_2_, while adding H_2_O_2_ directly to Hb solution only showed marginal cytotoxicity. These findings suggested that GOx played an important role in enhancing the Fenton reactions. The Calcein-AM/PI staining assay [50] was performed to further evaluate the anticancer effects of RBC@Hb@GOx NPs (Fig. 4c). The dead or alive cells were differentiated by the fluorescence color (red fluorescent spots for dead cells, green spots for living cells). It can be seen that in the presence of glucose, the Hb@GOx NPs and RBC@Hb@GOx NPs showed the highest red/green ratio, indicating the excellent capability of inhibiting the growth of cancer U87MG cells.

To quantify the intracellular ROS generated by the RBC@Hb@GOx NPs, 2′-7′-dichlorofuorescin diacetate (DCFH-DA) assay was used [51] which converted non-fluorescent DCFH-DA into fluorescent 2′-7′-dichlorofuorescin (DCF) when oxidized by ROS. In Fig. 4d, no obvious green fluorescence was observed in U87MG cells for the PBS group and Hb@GOx NPs or RBC@Hb@GOx NPs in the absence of glucose, suggesting insignificant ROS production. Notably, groups Hb@GOx NPs or RBC@Hb@GOx NPs with glucose induced a large amount of intracellular ROS as exhibited by the strong green fluorescence emission of DCF. Furthermore, the intracellular ROS level was determined via flow cytometry experiment. As shown in Fig. 4e, the fluorescent intensity of U87MG cells incubated with Hb@GOx NPs (33.57%) or RBC@Hb@GOx NPs (35.36%) in the presence of glucose was stronger than PBS group (4.38%), which indicated that both Hb@GOx NPs and RBC@Hb@GOx NPs were highly efficient in generating ROS. Similar amount of ROS was generated from Hb@GOx NPs and RBC@Hb@GOx NPs, meaning that the modification of the RBC membrane had a negligible effect on the catalytic performance. Together with the ESR experimental results, it can be concluded that in the presence of glucose, RBC@Hb@GOx NPs could be able to produce massive intracellular ROS to kill tumor cells.

### Evaluation of In Vitro Mitochondrial and Endoplasmic Reticulum Damage

**Fig. 5:**
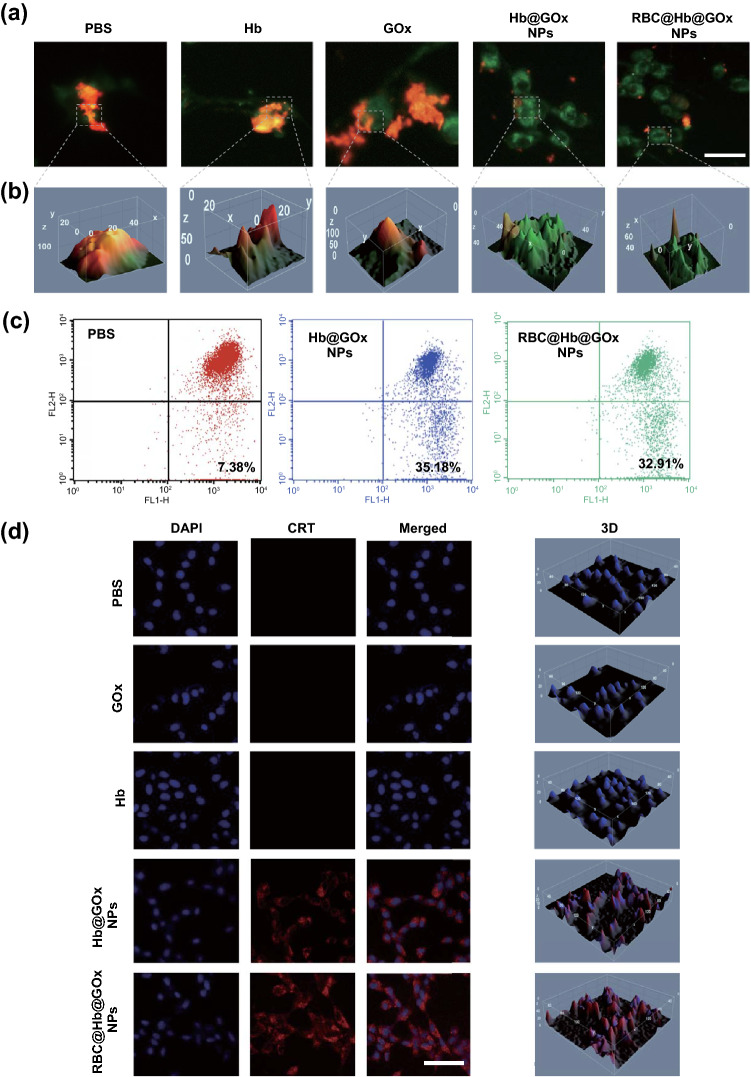
Mitochondrial and endoplasmic reticulum damage induced by RBC@Hb@GOx NPs. **a** CLSM images of JC-1 stained U87MG cells after different treatments. The fluorescence transition from red to green indicated significant mitochondrial damage. Scale bar: 50 μm. **b** 3D images of JC-1 stained U87MG cells after different treatments. **c** Evaluation of mitochondrial potential changes via flow cytometer test after different treatments. **d** Evaluation of CRT transferring via CRT staining assay after different treatments. Scale bar: 50 μm

We further investigated the mechanisms of ROS-mediated cell death. Mitochondria are central organelles and play a critical role in apoptosis in reacting to stress. Many factors induce cell apoptosis, including loss of mitochondrial membrane potential, release of cytochrome c (Cyto *c*), and caspase-activating protein. In the cell apoptosis progress, the loss of mitochondrial membrane potential is normally a signal of early mitochondrial damage, which can be investigated by JC-1 staining assay. It has been proved that JC-1 is an ideal fluorescent probe to detect mitochondrial membrane potential (∆Ψ*m*). When the mitochondrial membrane potential is high (normal mitochondria), JC-1 dyes aggregate in the mitochondrial matrix to form polymers called JC-1 aggregates, producing red fluorescence. While JC-1 aggregates are release from mitochondria matrix to cytoplasm to form JC-1 monomer, corresponding fluorescence transits from red to green, indicating that the mitochondrial membrane potential is low (damaged mitochondria) (Fig. S12). Thus, it is very convenient to detect mitochondrial membrane potential through observing the changes of fluorescent color and the relative ratio of red and green fluorescence [52, 53]. In this work, UMG87 cells incubated with different samples were stained with JC-1 dyes and analyzed via flow cytometry. As shown in Fig. 5a, b, the red fluorescence (normal mitochondria) of UMG87 cells in three groups including PBS, Hb, and GOx was observed. In contrast, the increase in green fluorescence intensity (damaged mitochondria) was obviously seen in groups treated with Hb@GOx NPs and RBC@Hb@GOx NPs with glucose (10 mM) after 20 min incubation. The Flow cytometry plots in Fig. 5c shows the percentage of mitochondrial membrane depolarized cells increased from 7.38% (control group) to 35.18% (Hb@GOx NPs with 10 mM glucose) and 32.91% (RBC@Hb@GOx NPs with 10 mM glucose), respectively.

As mentioned in previous studies, calreticulin (CRT) is rich in endoplasmic reticulum. Normally, ROS-based endoplasmic reticulum stress would induce the CRT transfer to the surface of plasma membrane (Fig. S13a). Besides, high mobility group protein B1 (HMGB1), as a highly conserved nuclear protein, is also released from the nuclear while the cells are dying [54]. Therefore, the CRT exposure and HMGB1 release were assessed to evaluate the intracellular oxidative stress in UMG87 cells. We evaluated the CRT exposure in UMG87 cells after incubating with PBS, Hb, GOx, Hb@GOx NPs, and RBC@Hb@GOx NPs (all groups treated with glucose solution, 10 mM) via immunofluorescence staining and CLSM analysis. As displayed in Fig. 5d, the largest number of CRT staining cells was detected in the Hb@GOx NPs and RBC@Hb@GOx NPs groups, indicating that these two groups could significantly induce the CRT translocation from the endoplasmic reticulum to cell surface due to the large amount of ROS generation. Next, the increased HMGB1 release in cell culture supernatant from the Hb@GOx NPs and RBC@Hb@GOx NPs-treated UMG87 cells was also detected by western blot analysis (Fig. S13b). The above findings revealed that the intracellular ROS generated by RBC@Hb@GOx NPs caused mitochondria damage and led to the eventual cell apoptosis.

### Evaluation the Biodistribution of RBC@Hb@GOx NPs In Vivo

**Fig. 6:**
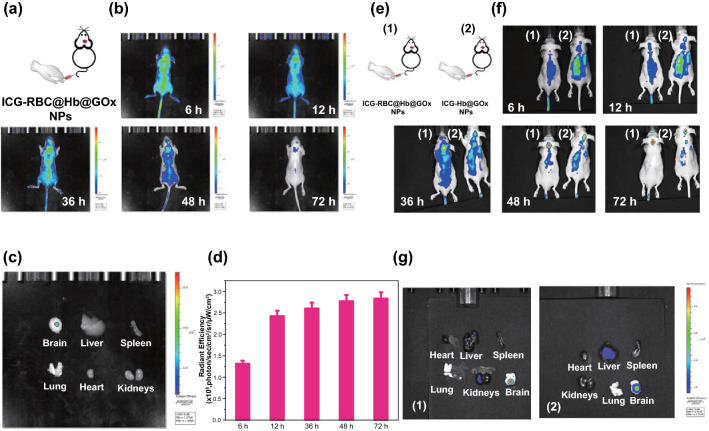
In vivo fluorescence imaging of orthotopic U87MG tumor-bearing mice for evaluating the transportability across the BBB. **a** Schematic illustration of injection of ICG-RBC@Hb@GOx NPs to orthotopic U87MG tumor-bearing mice. **b** Real-time fluorescence imaging of tumor-bearing mice after intravenous injection of ICG-RBC@Hb@GOx NPs. **c** Ex vivo fluorescent images of major organs and tumors at 72 h post-injection. **d** Semiquantitative fluorescence analysis in the tumor site at different time points after injection of ICG-RBC@Hb@GOx NPs. **e** Schematic illustration of injection of ICG-RBC@Hb@GOx and ICG-Hb@GOx NPs NPs to orthotopic U87MG tumor-bearing mice. **f** Real-time fluorescence imaging of tumor-bearing mice after intravenous injection of ICG-RBC@Hb@GOx NPs and ICG-Hb@GOx NPs. **g** Ex vivo fluorescent images. Data are shown as mean ± SD (*n* = 3)

Orthotopic U87MG gliomas (without fluorescence) bearing mice were constructed as animal models. ICG-loaded RBC@Hb@GOx NPs were injected intravenously to investigate their ability to cross the BBB (Figs. 6a and S14) [27]. In this study, we employed a passive targeting strategy. As shown in Fig. 6b, the fluorescence at the tumor site was increased after 12 h post-injection with RBC@Hb@GOx NPs. Interestingly, it maintained strong fluorescence signals even after 72 h. The ex vivo fluorescence images of major organs and brain (corresponding fluorescence intensity is shown in Fig. S15) were obtained at 72 h (Fig. 6c). There was an obvious fluorescence signal in the brain, while no apparent fluorescence could be observed in the other organs. Combing the above results with the fluorescence intensity at each time point (Fig. 6d) and staining experiments of the slice from different organs (Fig. S16), we could conclude that the RBC@Hb@GOx NPs crossed the BBB and exhibited notable tumor accumulation and prolonged retention properties. Compared to receptor-mediated transcytosis, the delivery efficiency was significantly increased.

To further investigate the role of RBC membrane in crossing the BBB, we synthesized ICG-loaded RBC@Hb@GOx NPs and Hb@GOx NPs to compare their accumulation in tumors (Fig. 6e). From Fig. 6f, we found that the ICG-Hb@GOx NPs-treated group also exhibited strong fluorescence after 36 h post-injection, whereas the fluorescence intensity in the ICG-RBC@Hb@GOx NPs-treated group was considerably higher (Fig. S17a, b). The results showed that the RBC membrane was not the essential component but rather a facilitator in BBB crossing. This might be because that the RBCs membrane improved the circulation time and made the NPs softer and easier to deform, providing higher chances for the NPs to transverse the BBB. Similarly, the fluorescence imaging and intensity for major organs were tested after 72 h post-injection (Figs. 6g and S17c) and TUNEL staining from different organs (Fig. S18), showing that obvious fluorescence signals could be observed in both two groups. Therefore, we believe that in this case, the long circulating time and the small size are the contributing factors for the NPs to cross BBB and accumulate at the tumor site.

### Evaluation of In Vivo CDT of Orthotopic GBM Tumor

Encouraged by the satisfactory BBB transportability in vivo, we further investigated the therapeutic efficacy of RBC@Hb@GOx NPs. The mice bearing orthotopic GBM tumor with self-fluorescence were constructed. IVIS^®^ imaging system was used to track the tumor response to treatment. Firstly, we did a short-term survival experiment in mice (Fig. S19a). PBS (control group), GOx, and RBC@Hb@GOx NPs were administrated by tail vein injection to evaluate the therapeutic performances, respectively. As shown in Fig. S19b, during 15-day treatment, the fluorescence intensity in control and GOx groups showed significant increase, indicating that no therapeutic effect on the brain tumor. In contrast, for the group treated with RBC@Hb@GOx NPs, fluorescence intensity increased slowly and the area with fluorescence was much smaller compared to the other two groups, which implied that the RBC@Hb@GOx NPs was able to suppress the tumor growth. The therapeutic effect was further confirmed by H&E and Congo red staining (Fig. S19c). These staining images indicated an extensive cell apoptosis in GBM in the RBC@Hb@GOx NPs-treated group. Besides, the whole brain was also sliced from mice after 15 days (Fig. S20a). It was apparent that the satisfactory tumor suppression effects came from the highly toxic hydroxyl radicals produced by the sequential biological/chemical-catalytic reactions by the RBC@Hb@GOx NPs. The as-produced toxic hydroxyl radicals then killed the cancer cells in a mitochondria-mediated apoptosis pathway.

**Fig. 7:**
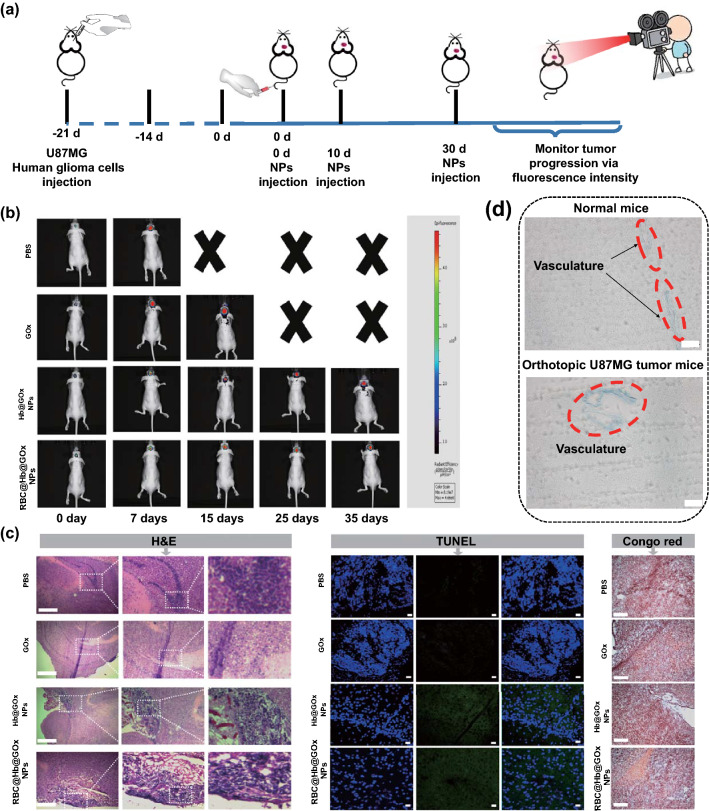
In vivo therapeutic efficacy of Hb@GOx NPs and RBC@Hb@GOx NPs in orthotopic glioma-bearing mice. **a** Schematic illustration of long-term survival experiment in orthotopic U87MG tumor-bearing mice with PBS, GOx, Hb@GOx NPs, and RBC@Hb@GOx NPs injection. **b** Representative bioluminescent images of orthotopic U87MG tumor-bearing mice in different groups in 35-day treatments. **c** Orthotopic U87MG tumor in the brain after H&E (scale bar: 50 μm), TUNEL (scale bar: 20 μm), and Congo red staining (scale bar: 50 μm) after treatments. **d** EB staining of brain sections after treatment. Scale bar: 50 μm

A long-term survival experiment was conducted in mice to compare the therapeutic effect of Hb@GOx NPs and RBC@Hb@GOx NPs as outlined in Fig. 7a. It is clearly seen from Fig. 7b that both Hb@GOx NPs- and RBC@Hb@GOx NPs-treated groups significantly suppressed the growth of U87MG glioblastoma tumor. The tumor suppression efficiency was quantified by measuring the changes of the fluorescence intensity at the tumor sites. As presented in Fig. S20b, quantitative analysis showed that when treated with PBS or GOx, the orthotopic brain tumor grew rapidly. However, the tumor treated with Hb@GOx NPs and RBC@Hb@GOx NPs maintained relatively constant size. As shown in Fig. 7c, the H&E staining experiments of brains tumor tissue were obtained from control group (PBS)-treated mice and mice co-treated with GOx, Hb@GOx NPs, and RBC@Hb@GOx NPs. In control group, the H&E staining experiments presented large tumor cell populations with higher instance of actively dividing nuclear morphology. For the treated groups, H&E stains shown that there was an obvious area of dead cells with radial propagations in orthotopic U87MG tumor. These results were in close agreement with TUNEL staining and Congo red staining. Finally, typical Evans blue (EB) stained brains in Fig. 7d showed that both normal mice and orthotopic U87MG tumor-bearing mice maintained an intact structure of BBB, because the EB could only be detected in brain vascular, further validating that our NPs possessed an excellent transportability of BBB. Finally, the slight changes in body weight (Fig. S21a) and the survival rate (Fig. S21b) indicated that RBC@Hb@GOx NPs possessed a remarkable biocompatibility.

Besides, the blood biochemical data, including red blood cells (RBC), white blood cells (WBC), hemoglobin (HGB), hematocrit (HCT), mean corpuscular volume (MCV), mean corpuscular hemoglobin concentration (MCHC), platelets (PLT), and mean corpuscular hemoglobin (MCH), liver and kidney function test including alanine transaminase (ALT), aspartate transaminase (AST), albumin (ALB), creatinine (CREA), blood urea nitrogen (BU), and uric acid (UA) were applied to evaluate the in vivo toxicity of RBC@Hb@GOx NPs (Fig. S22). There was no apparent discrepancy can be observed from all groups, what is more, no significant pathological changes of the major organs from all experimental groups were observed by H&E stained organ slices (Fig. S23), demonstrating the excellent biocompatibility of RBC@Hb@GOx NPs. These results showed that the RBC@Hb@GOx NPs as a powerful CDT nanoagent could efficiently inhibit the growth GBM and elongate the survival time.

## Conclusions

In summary, we developed a new type of CDT nanoagent for GBM treatment. The natural Hb and GOx were employed as highly efficient Fenton catalysts. The excellent biocompatibility and biodegradability greatly reduced the toxicity of the nanomaterials and improved the safety of CDT. With facile assembling and crosslinking techniques, Hb and GOx were formulated into protein superstructures. They could be utilized as self-delivery entities, thereby eliminating the need of conventional drug carriers. RBC membranes were coated on the protein superstructures to reduce the immunogenicity and enhance the circulation time, which facilitated the delivery of the NPs to cross BBB/BTB. We showed that the RBC@Hb@GOx NPs with the size of 51.55 nm could accumulate and retain at the GBM tumor site. More importantly, we successfully demonstrated in both in vitro and in vivo studies that the NPs could elicit powerful anti-tumor efficacy by generating ROS to induce mitochondria-mediated apoptosis. The RBC@Hb@GOx NPs thus presented a promising therapeutic direction for GBM treatment. We envision that the strategy could also be useful for other primary and metastatic brain tumors.

## Electronic supplementary material

Below is the link to the electronic supplementary material.

Supplementary material 1 (PDF 2142 kb)
