# Supplementary material for: Self-Assembly Protein Superstructures as a Powerful Chemodynamic Therapy Nanoagent for Glioblastoma Treatment
Source: Nanomicro Lett. 2020 Jul 15;12:151. doi: 10.1007/s40820-020-00490-6 (PMC7770858; doi:10.1007/s40820-020-00490-6)
Supplement: Supplementary file 1 — Supplementary material 1 (PDF 2142 kb) [file 40820_2020_490_MOESM1_ESM.pdf]

Supporting Information for

Self-Assembly Protein Superstructures as a Powerful Chemodynamic Therapy Nanoagent for Glioblastoma Treatment

Tao Zheng[1,] *, Wentao Wang[1], Jon Ashley[1], Ming Zhang[1,] *, Xiaotong Feng[1], Jian Shen[2, 3], Yi Sun[1,] *

[1]Department of Health Technology, Technical University of Denmark, Kongens Lyngby DK-2800, Denmark

[2]Jiangsu Collaborative Innovation Center for Biomedical Functional Materials, School of Chemistry and Materials Science, Nanjing Normal University, Nanjing 210023, People's Republic of China

[3]Key Laboratory of High Performance Polymer Material and Technology of Ministry of Education, School of Chemistry and Chemical Engineering, Nanjing University, Nanjing, 210023, People's Republic of China

*Corresponding authors. E-mail: taozhe@dtu.dk (Tao Zheng); mzhan@dtu.dk (Ming Zhang); suyi@dtu.dk (Yi Sun)

S1 Supplementary Methods

Chemicals. All of the chemicals were used without further purification. All agents were obtained from Sigma-Aldrich. All procedures for animal experiments were handled under the guidelines approved and supervised by the ethics committee of Nanjing University and Nanjing Normal University.

Apparatus. The infrared spectrum was performed on a (FT-IR) Nexus 670 FTIR type (Nicolet). UV-Vis spectra and FL spectra were recorded using a Tecan Spark® multimode plate reader. The surface composition and element analysis of the samples were recorded using X-ray photoelectron spectroscopy (XPS, EscaLab-250, Thermo, USA). Transmission electron microscopy (TEM) analyses of samples were carried out on a Tecnai G20 operating. ESR spectrum of sample was measured by using Bruker EMXplus Spectrometer System. Zeta potential was conducted using Malvern DLS Zetasizer. The fluorescent images of cells were acquired by Laser Scanning Confocal Microscope (LSCM, TI-E-AIR, Nikon, Japan). The flow cytometry experiments was conducted via using Becton-Dickinson FACS Calibur (USA). In vivo fluorescence imaging to evaluate transportability of across the BBB and antiglioma efficiency *in vivo* of NPs was observed by bioluminescence IVIS® imaging equipped with LivingImage™ software (Xenogen). TUNEL, H&E and Congo red staining was determined via inverted biological microscope (Olympus BX43 microscope, Japan).

S2 Supplementary Figures

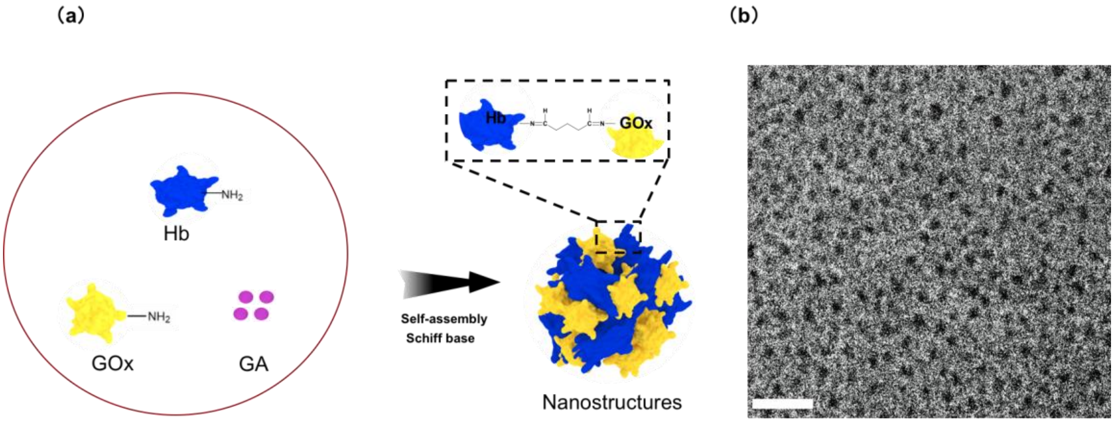

Fig. S1 Schematic illustration of synthetic route of Hb@GOx NPs. **a** Synthetic route of Hb@GOx NPs. **b** TEM images of as-fabricated Hb@GOx NPs. Scale bar: 100 nm

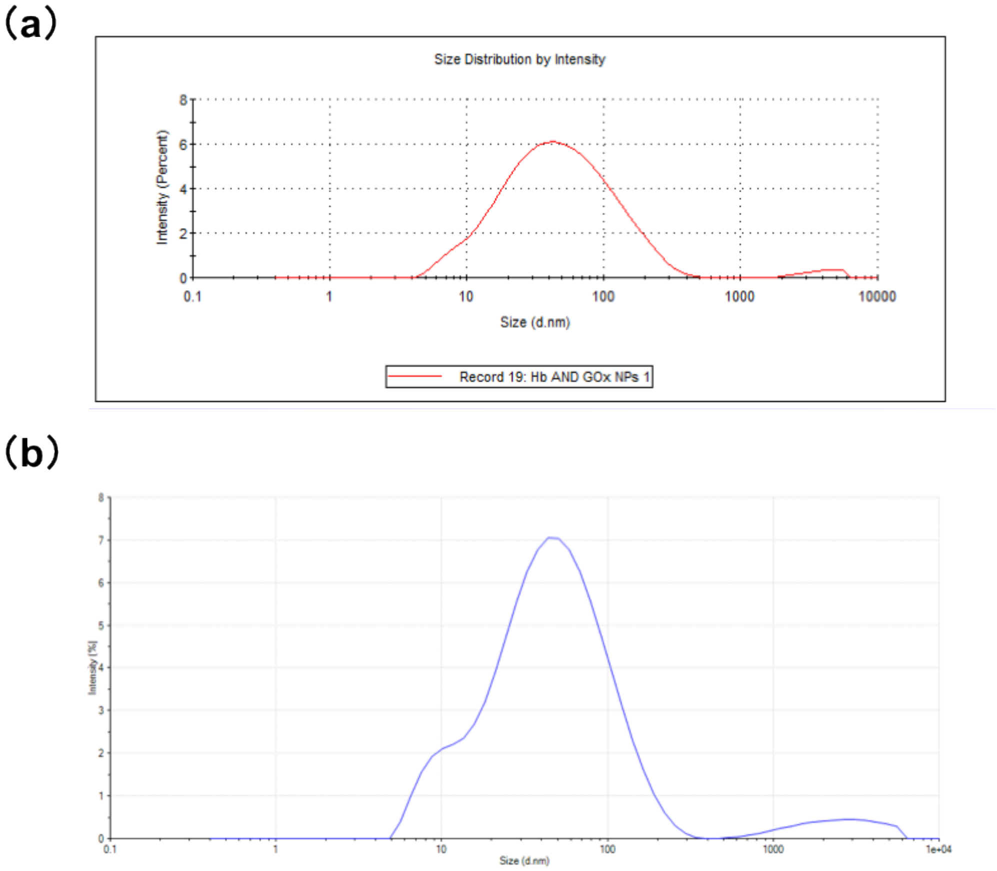

Fig. S2 Characterization of as-fabricated Hb@GOx NPs and RBC@Hb@GOx NPs. **a** Size of as-fabricated Hb@GOx NPs (24 h) determined by DLS. **b** Size of as-fabricated RBC@Hb@GOx NPs (24 h) determined by DLS

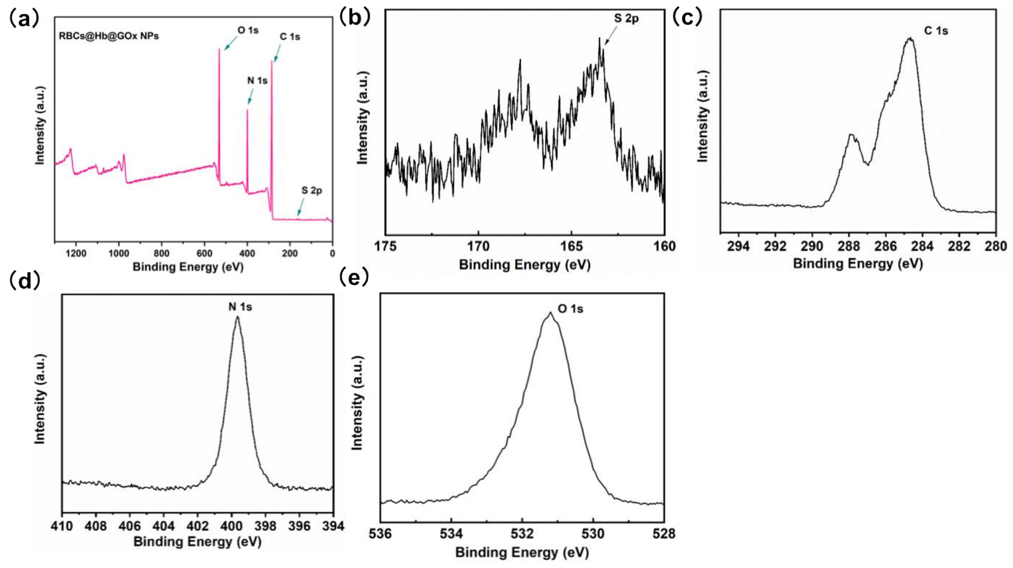

Fig. S3 XPS characterization of RBC@Hb@GOx NPs (without glucose). **a** XPS of RBC@Hb@GOx NPs without contacting with glucose. High-resolution XPS spectra of **b** S 2p, **c** C 1s, **d** N 1s, and **e** O 1s

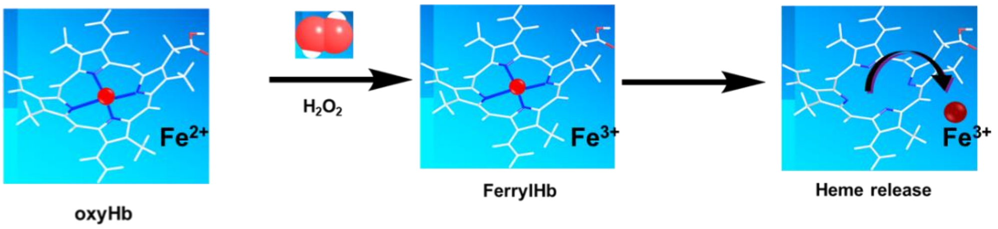

Fig. S4 Schematic illustration of the Fe^{2+} releasing from heme when contacting with H_2O_2

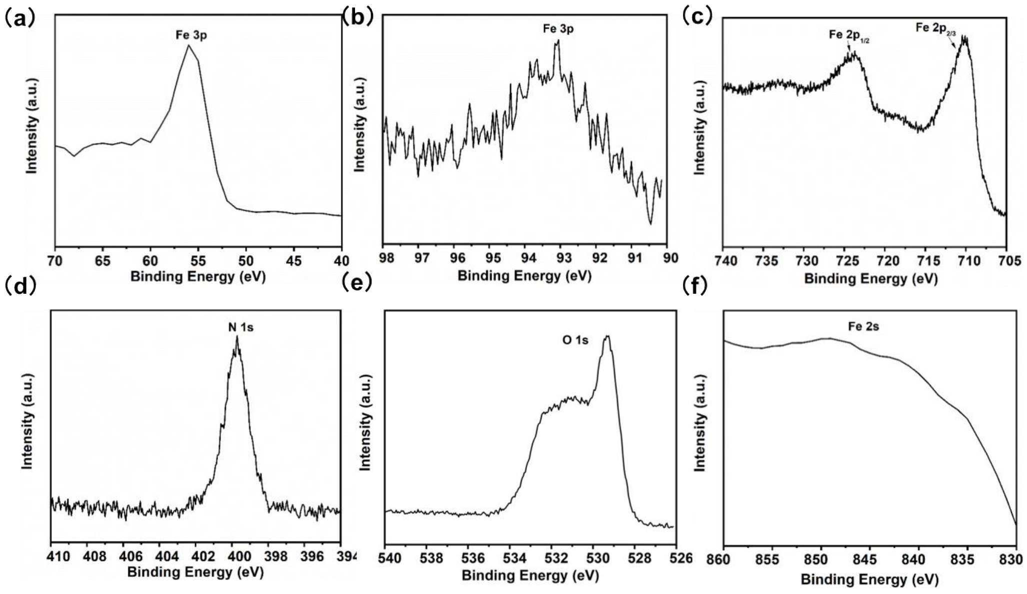

Fig. S5 XPS characterization of RBC@Hb@GOx NPs (with glucose). High-resolution XPS spectra of **a, b** Fe 3p, **c** Fe 2p, **d** N 1s, **e** O 1s, **f** Fe 2s

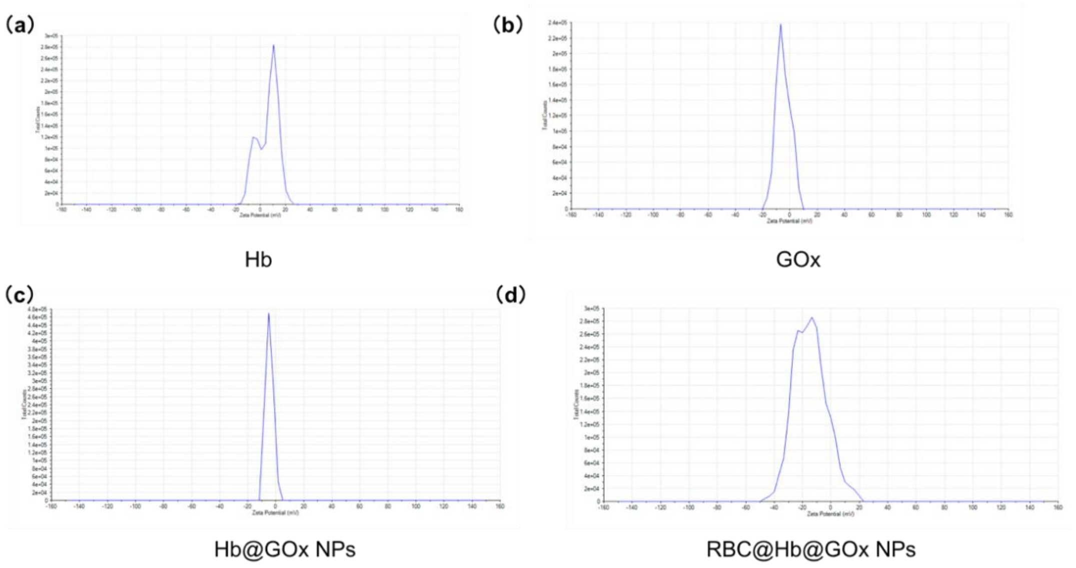

Fig. S6 Zeta potential of **a** Hb, **b** GOx, **c** Hb@GOx NPs, **d** RBC@Hb@GOx NPs

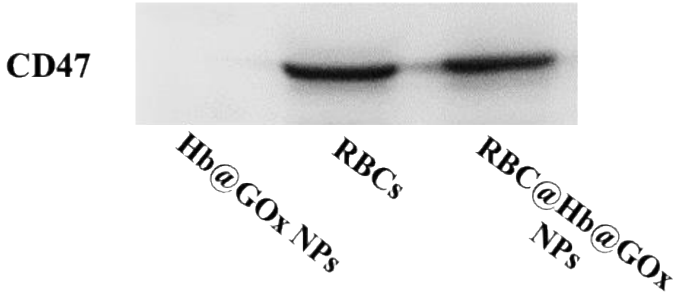

Fig. S7 Western blotting assay for analyzing CD47 protein. Group 1 was Hb@GOx NPs, group 2 was RBCs, and group 3 was RBCs@ Hb@GOx NPs

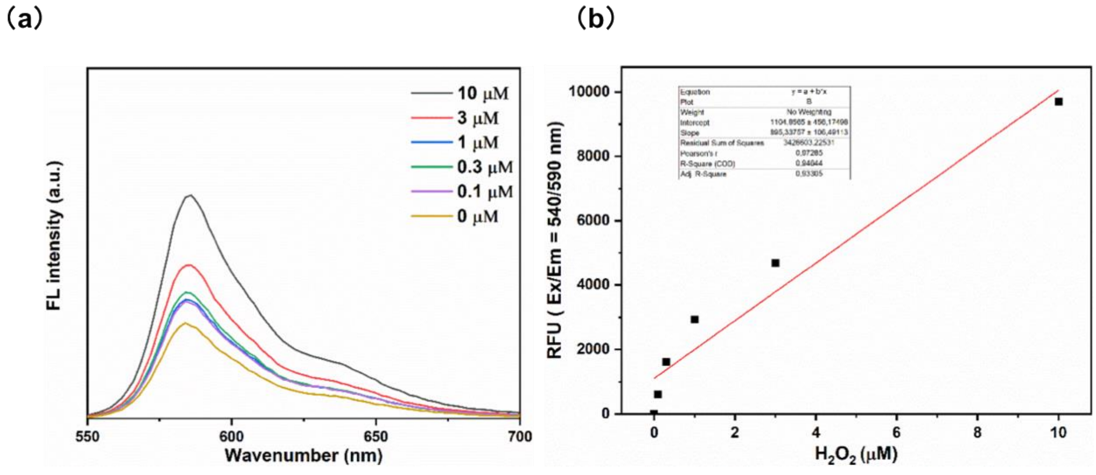

Fig. S8 Evaluation of catalytic performance of RBC@Hb@GOx NPs *in vitro*. **a** Changes of fluorescence intensity of Amplex red with different concentrations of H_2O_2. **b** Liner curve of H_2O_2 concentration-dependent fluorescence intensity changes of Amplex red

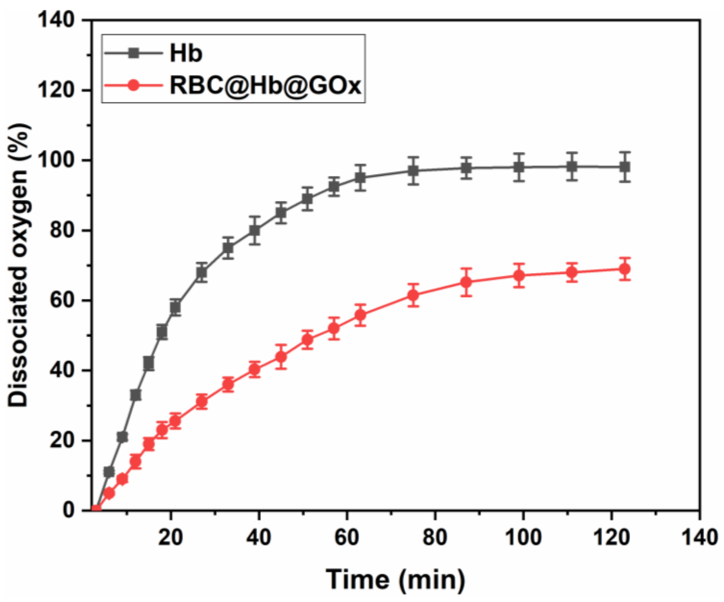

Fig. S9 Dissolved O_2 of Hb and RBC@Hb@GOx NPs

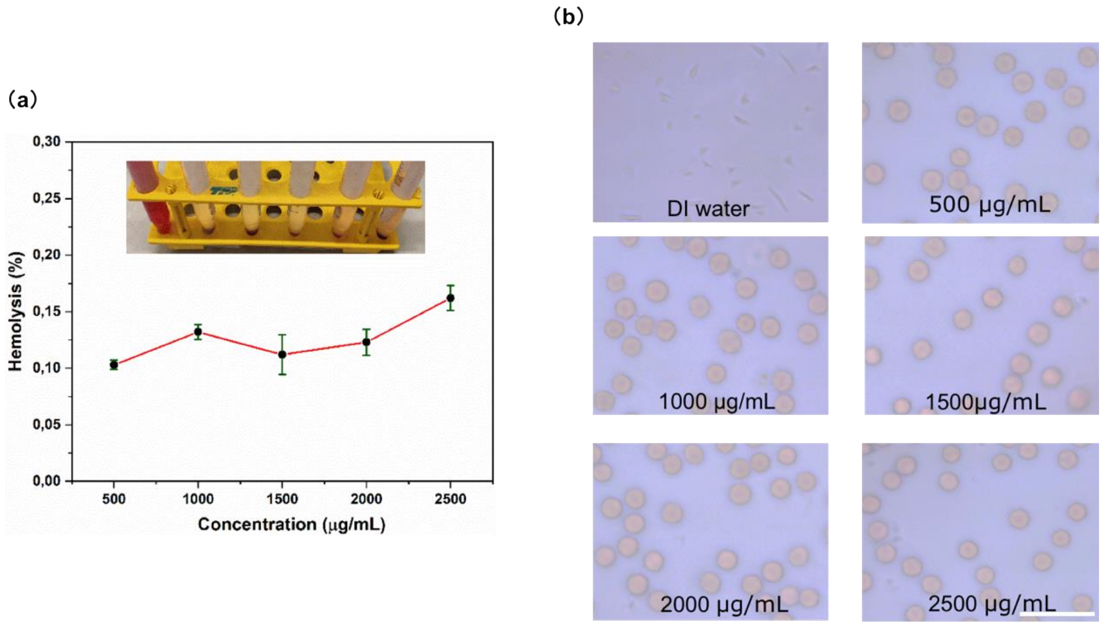

Fig. S10 a Hemolysis of RBC@Hb@GOx NPs solution at various concentrations. Inset: the mixtures were centrifuged to detect the presence of hemoglobin in the supernatants visually. **b** The morphology of red blood cells treated with RBC@Hb@GOx NPs at different concentrations. Scale bar: 50 μm. Data are presented as mean ± s.d. (n = 3).

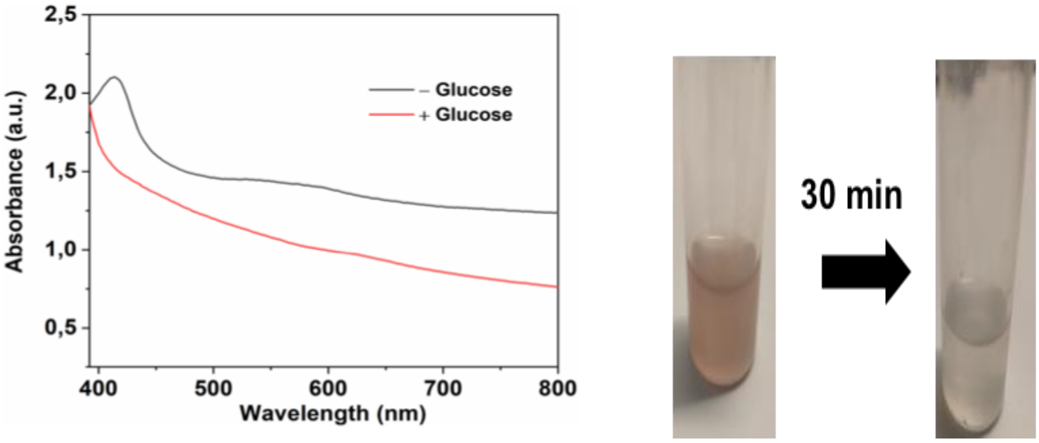

Fig. S11 Degradation of RBC@Hb@GOx NPs in glucose solution (10 mM). Inset is a photograph of the two groups

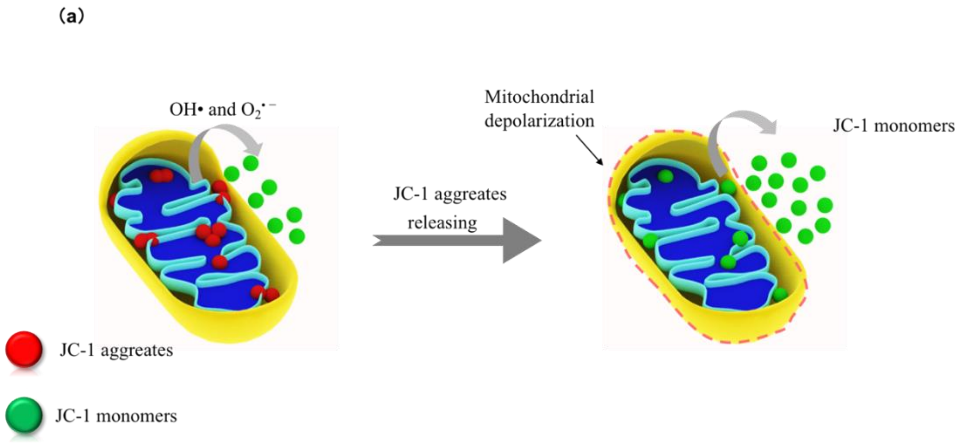

Fig. S12 Evaluation of *in vitro* mitochondrial damage. The mechanism of probe of JC-1 responding to mitochondrial membrane potential.

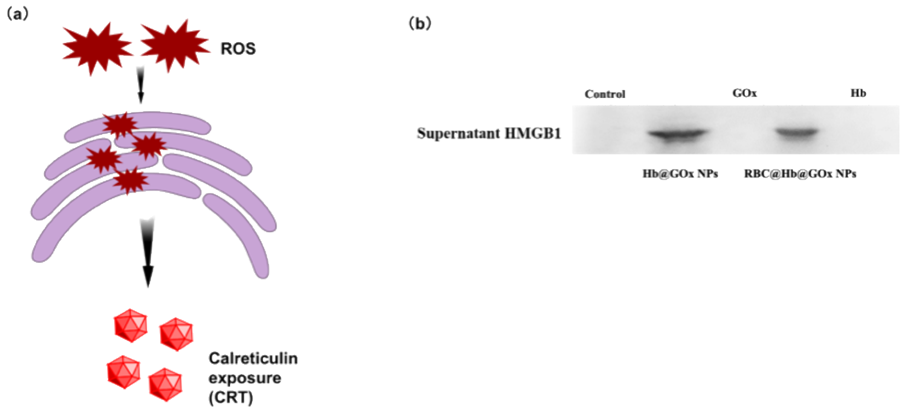

Fig. S13 Evaluation of *in vitro* endoplasmic reticulum damage**. a** Mechanism of probe of CRT staining responding to ROS-based endoplasmic reticulum stress. **b** Western blots of HMGB1 of different groups

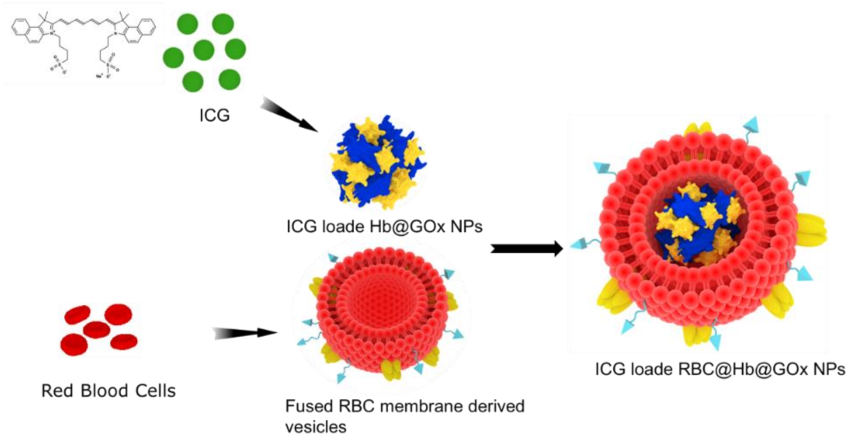

Fig. S14 Schematic illustration of ICG loaded RBC@Hb@GOx NPs were injected intravenously to investigate their ability to cross the BBB

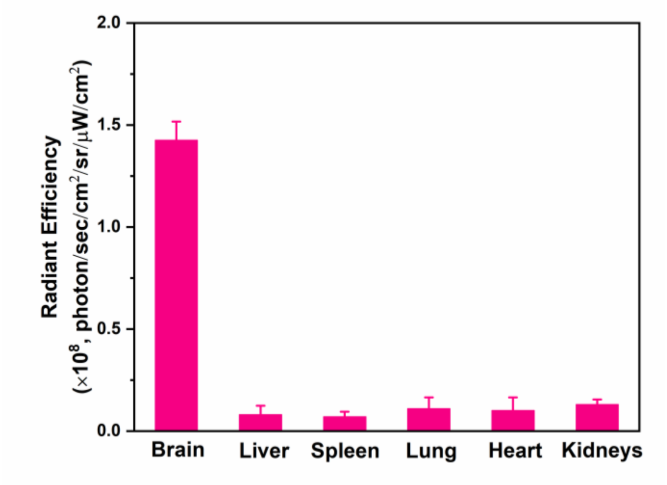

Fig. S15 Evaluation the biodistribution of RBC@Hb@GOx NPs *in vivo*. Ex vivo fluorescence intensity in major organs and brain after 72 h injection

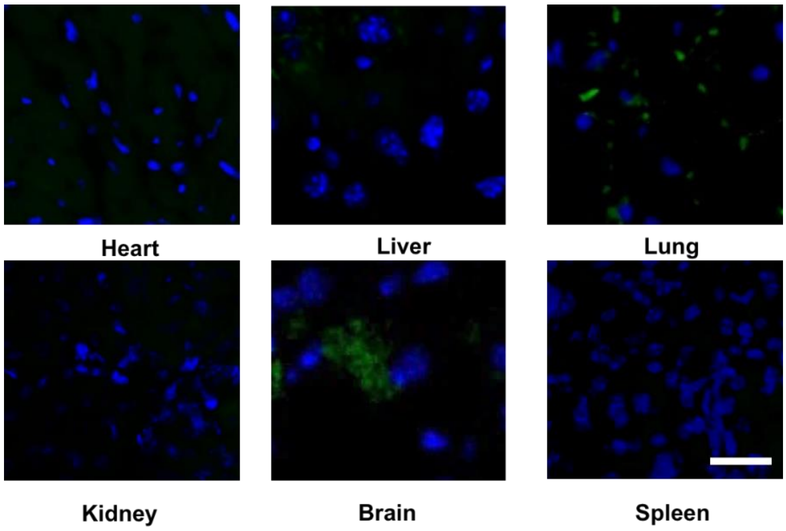

Fig. S16 Evaluation the biodistribution of RBC@Hb@GOx NPs *in vivo*. Biodistribution of RBC@Hb@GOx NPs in different organs. Scale bar: 20 µm.

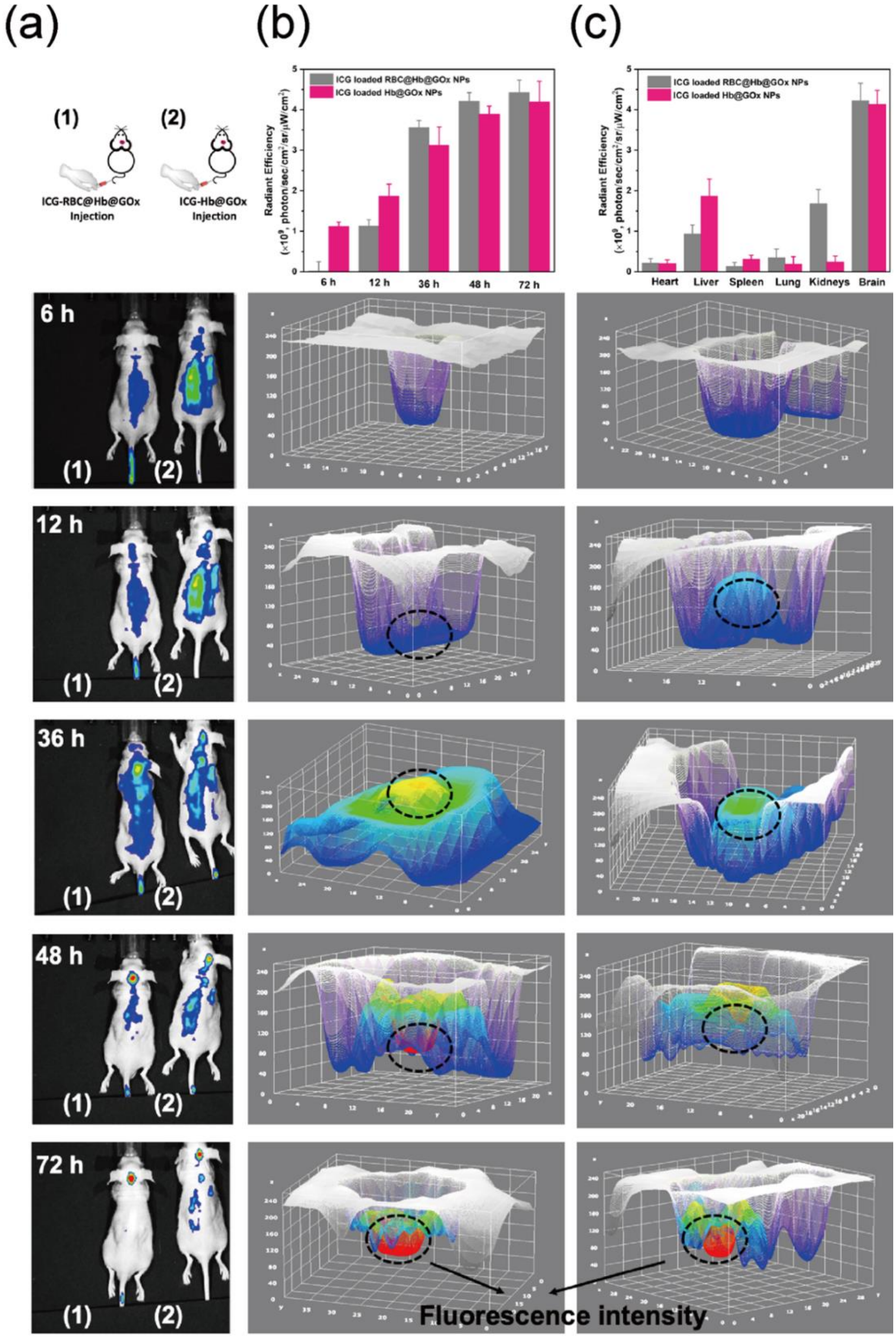

Fig. S17 Evaluation the biodistribution of RBC@Hb@GOx NPs *in vivo*. **a** The 3D view of fluorescence imaging in the tumor site at different time points after injection of ICG- Hb@GOx NPs and ICG-RBC@Hb@GOx NPs. **b** Semiquantitative fluorescence analysis in the tumor site at different time points after injection of ICG-Hb@GOx NPs and ICG-RBC@Hb@GOx NPs. **c** Ex vivo fluorescence intensity in major organs and brain after 72 h injection in ICG-Hb@GOx NPs and ICG-RBC@Hb@GOx NPs treated groups

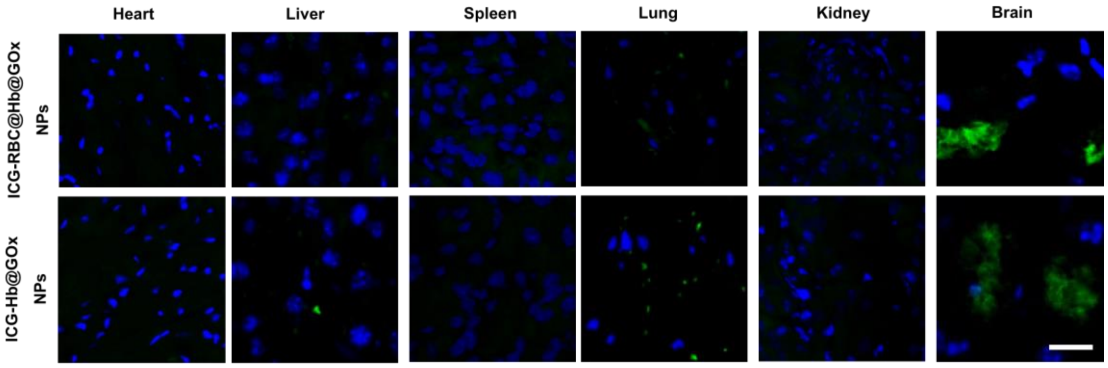

Fig. S18 Evaluation the biodistribution of Hb@GOx NPs and RBC@Hb@GOx NPs *in vivo*. Biodistribution of Hb@GOx NPs and RBC@Hb@GOx NPs in different organs. Scale bar: 20 μm

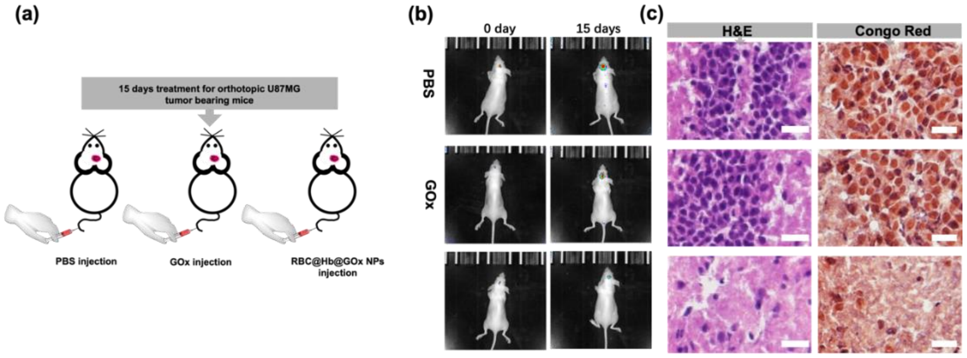

Fig. S19 *In vivo* therapeutic efficacy of Hb@GOx NPs and RBC@Hb@GOx NPs in orthotopic glioma-bearing mice. **a** Schematic illustration of short-term survival experiment in orthotopic U87MG tumor bearing mice with ICG-RBC@Hb@GOx NPs injection. **b** Representative bioluminescent images of orthotopic U87MG tumor bearing mice in different groups after 15-days treatment. **c** H&E staining and Congo red staining of brain sections of orthotopic glioma-bearing mice in all groups. Scale bar: 50 μm

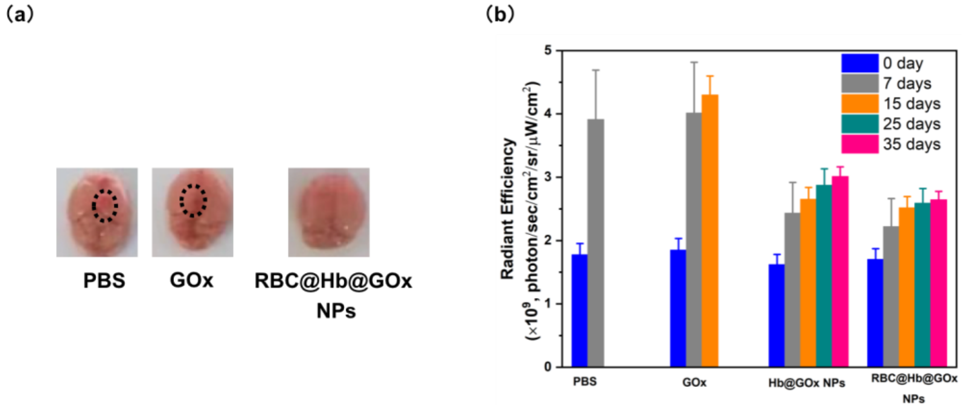

Fig. S20 Evaluation of *in vivo* CDT of Orthotopic GBM tumor. **a** The whole brain sliced after 15 days treatment. **b** Semiquantitative fluorescence analysis in the tumor site at different time points after injection of RBC@Hb@GOx NPs

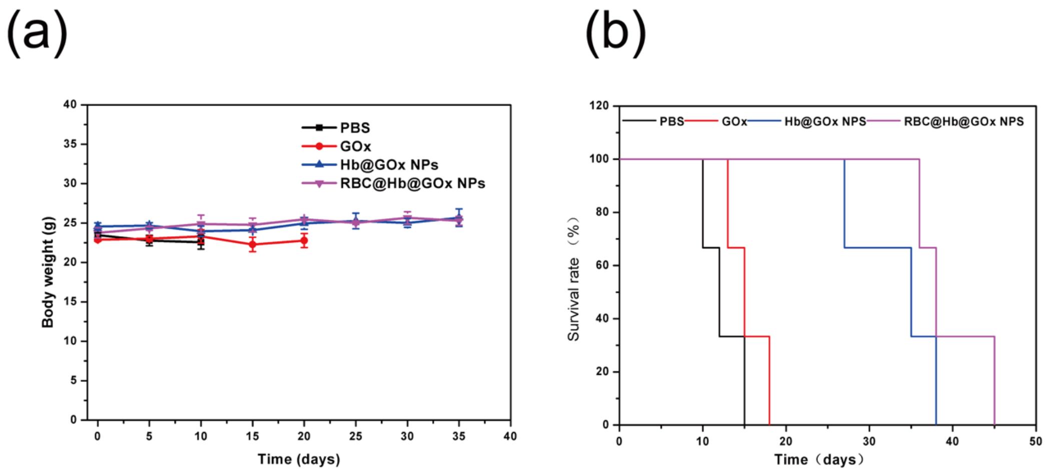

Fig. S21 a Body weight changes. **b** Survival rate of orthotopic brain tumor-bearing mice of different groups with different treatments

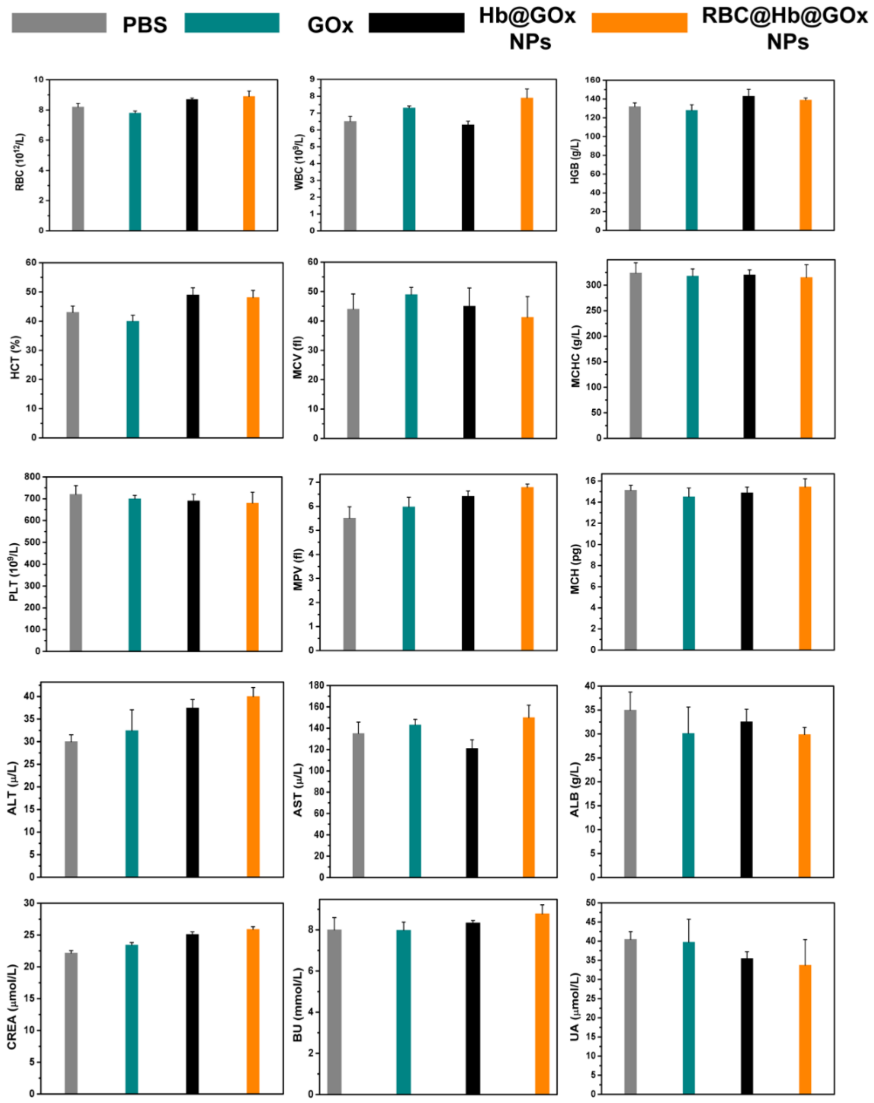

Fig. S22 Haematological parameter of the mice treated with different groups at the 35 days post-injection

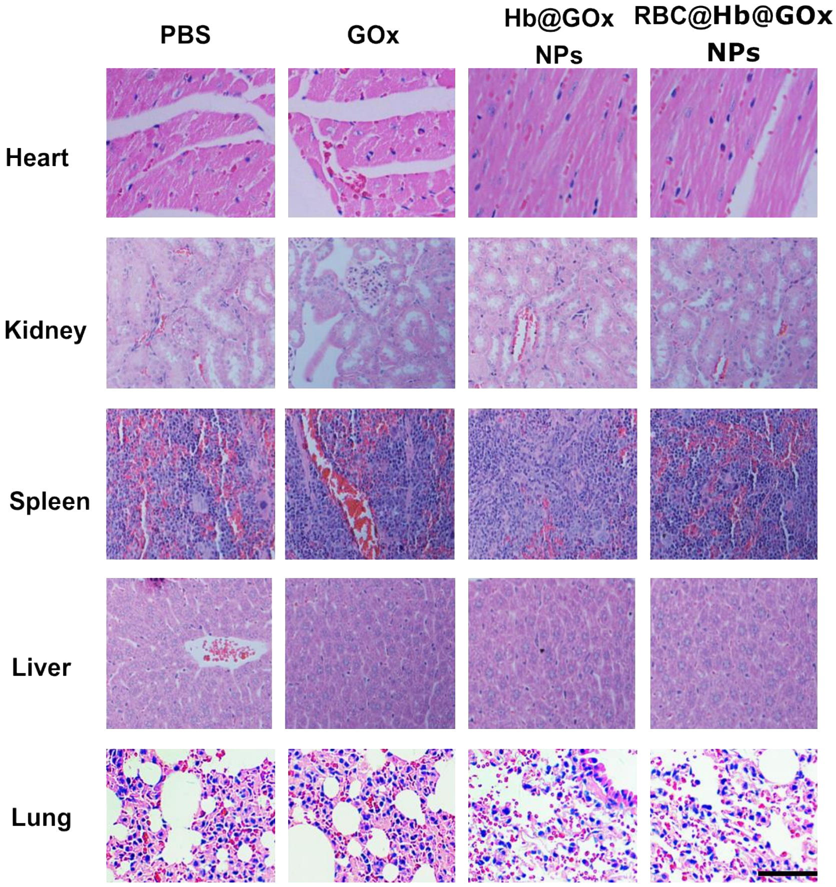

Fig. S23 Hematoxylin and eosin (H&E) stained histological images of dissected major organs treated with different conditions after 35 days. Scale bar: 50 μm. Data is shown as mean values ± s.d. (n = 3)
